# Liver transplantation at the extreme: Analysis of outcomes and predictors of futility in patients with acute-on-chronic liver failure grade 3 on maximal life support

**DOI:** 10.1016/j.jhepr.2026.101959

**Published:** 2026-08-05

**Authors:** Andrew Melehy, Asara Thepbunchonchai, Benjamin V. Tran, Ruben Hernaez, Dominic Amara, Samer Ebaid, Daniela Markovic, Fady M. Kaldas, Igor Barjaktarevic, Victor Xia, Constantine J. Karvellas, Saro Khemichian, Lance L. Stein, Kirti Shetty, Christina C. Lindenmeyer, Justin R. Boike, Douglas A. Simonetto, Robert S. Rahimi, Prasun K. Jalal, Manhal Izzy, Michael S. Kriss, Gene Y. Im, Ming V. Lin, Janice H. Jou, Brett E. Fortune, George Cholankeril, Alexander Kuo, Vinay Sundaram, Multi-Organ Dysfunction and Evaluation for Liver Transplantation (MODEL) Consortium, Atef Al Attar, Kambiz Kosari, Richard Garcia, Gevork Salmastyan, William Cranford, Preet Patel, Pei Xue, Soumya Mishra, Madison Parks, Gianina Flocco, Jing Gao, Tiffany Wu, Priya Thanneeru, Vikrant Reddy, Jing Gao, Mariana Hurtado, Islam Mohamed, Ross Vyhmeister, Christine R. Lopez, Braidie Campbell, Adam C. Winters, Mary Ann Simpson, Ronald W. Busuttil, Douglas G. Farmer, Vatche G. Agopian

**Affiliations:** 20 Departement Hospitalo-Universitaire, Hepatinov, Villejuif, France; 1 Dumont-UCLA Transplant and Liver Cancer Centers, Department of Surgery, David Geffen School of Medicine at University of California, Los Angeles, CA, USA; 2 Section of Gastroenterology, Michael E. DeBakey Veterans Affairs Medical Center, Houston, TX, USA; 3 VA HSR&D Center for Innovations in Quality, Effectiveness and Safety, Michael E. DeBakey VA Medical Center, Houston, TX, USA; 4 Section of Gastroenterology and Hepatology, Department of Medicine, Baylor College of Medicine, Houston, TX, USA; 5 Department of Critical Care Medicine and Division of Gastroenterology (Liver Unit), University of Alberta, Edmonton, AB, Canada; 6 Division of Gastrointestinal & Liver Disease, Keck Hospital at University of Southern California, Los Angeles, CA, USA; 7 Piedmont Transplant Institute, Piedmont Atlanta Hospital, Atlanta, GA, USA; 8 Division of Gastroenterology & Hepatology, University of Maryland School of Medicine, Baltimore, MD, USA; 9 Department of Gastroenterology, Hepatology and Nutrition, Cleveland Clinic, Cleveland, OH, USA; 10 Division of Gastroenterology and Hepatology, Department of Medicine, Northwestern University, Chicago, IL, USA; 11 Division of Gastroenterology and Hepatology, Mayo Clinic, Rochester, MN, USA; 12 Baylor University Medical Center, Division of Hepatology. Baylor Scott and White Hospital, Dallas, TX, USA; 13 Division of Gastroenterology, Hepatology and Nutrition, Vanderbilt University, Nashville, TN, USA; 14 Department of Medicine, University of Colorado, Aurora, CO, USA; 15 Recanati/Miller Transplantation Institute, Icahn School of Medicine at Mount Sinai, New York, NY, USA; 16 Division of Transplant and Hepatobiliary Diseases, Department of Surgery, Lahey Hospital and Medical Center, Burlington, MA, USA; 17 Medicine, Division of Gastroenterology, Oregon Health and Science University, Portland, OR, USA; 18 Division of Hepatology, Department of Medicine, Montefiore Medical Center, Bronx, NY, USA; 19 Karsh Division of Gastroenterology and Hepatology, Comprehensive Transplant Center, Cedars-Sinai Medical Center, Los Angeles, CA, USA

**Keywords:** Acute-on-chronic liver failure, Liver transplant, Maximal life support

## Abstract

**Background & Aims:**

Although patients with grade 3 acute-on-chronic liver failure (ACLF-3), have high mortality, there is a subset requiring maximal life support. We report post liver transplant (LT) outcomes and identify factors associated with futility in extreme ACLF-3 (eACLF-3) recipients, all requiring intubation, vasopressors, and dialysis at LT.

**Methods:**

We identified adult eACLF-3 LT recipients between January 2010 and December 2021. Pre-LT characteristics were compared among eACLF-3 recipients with and without the futile outcome (1-year or in-hospital mortality), predictors of futility were identified, and a logistic regression model was evaluated using fivefold cross-validation. Findings were validated using the Multi-Organ Dysfunction and Evaluation for Liver Transplantation (MODEL) Consortium.

**Results:**

Of 1,608 adult LT recipients at our center, 177 (11%) were eACLF-3. Thirty-five (20%) eACLF-3 recipients experienced futility. Predictors of futility were previous upper abdominal surgery (odds ratio [OR] = 4.6, *p* = 0.002), sepsis within 14 days before LT (OR = 5.9, *p* = 0.013), intubation because of pneumonia or acute respiratory distress syndrome (OR = 3.5, *p* = 0.010), worsening PaO_2_:FiO_2_ ratio (OR = 3.9, *p* = 0.008), and pre-LT base deficit (OR = 1.27, *p* = 0.007). Futility was predicted with an AUROC of 0.74 (SD 0.15). When compared with the MODEL Consortium, our cohort had improved 3-, 6-, and 12-month survivals of 89%, 85%, and 80%, compared with 83%, 79%, and 70%, respectively (*p* = 0.048); however overall 5-year survival was similar (63% *vs*. 59%, *p* = 0.14).

**Conclusions:**

We report the largest single-center experience of LT for patients with eACLF-3, demonstrating acceptable survival despite high acuity, validated in the MODEL Consortium dataset. Prospective validation of the predictive model and identified risk factors is necessary, and may aid in appropriate selection of patients with ACLF-3 requiring maximal life support before LT.

**Impact and implications:**

There has been persistent interest in the liver transplant community regarding transplant for acute-on-chronic liver failure grade 3 (ACLF-3) recipients. In this work, we report on a large single-center experience transplanting ACLF-3 recipients, specifically investigating an extreme subset of these patients who were all mechanically ventilated, receiving vasopressors, and receiving hemodialysis at time of transplant. We characterized their outcomes, identifying clear risk factors associated with futility in this hyperacute subgroup and demonstrating that with appropriate selection, even patients in this extreme subgroup can experience acceptable 5-year post-transplant survival. Although we could not externally validate our model because of data availability, the obtained external patient cohort of patients with extreme ACLF-3 broadly validates our findings. We feel that these results represent an important contribution to the liver transplant community given concerns about transplanting this hyperacute subpopulation. These results may help guide physicians in selecting appropriate candidates for transplant.

## Highlights

• LT recipients with ACLF-3 on maximal life support represent a unique, high-acuity transplant population.
• One-year mortality is 20%, but long-term survival is acceptable among patients surviving the first year.
• Sepsis within 2 weeks before transplant is a risk factor for futility in this high-acuity subpopulation.
• Intubation because of pneumonia or acute respiratory distress syndrome and worsening PaO_2_:FiO_2_ ratio predict futility.

## Graphical abstract

**Figure:**
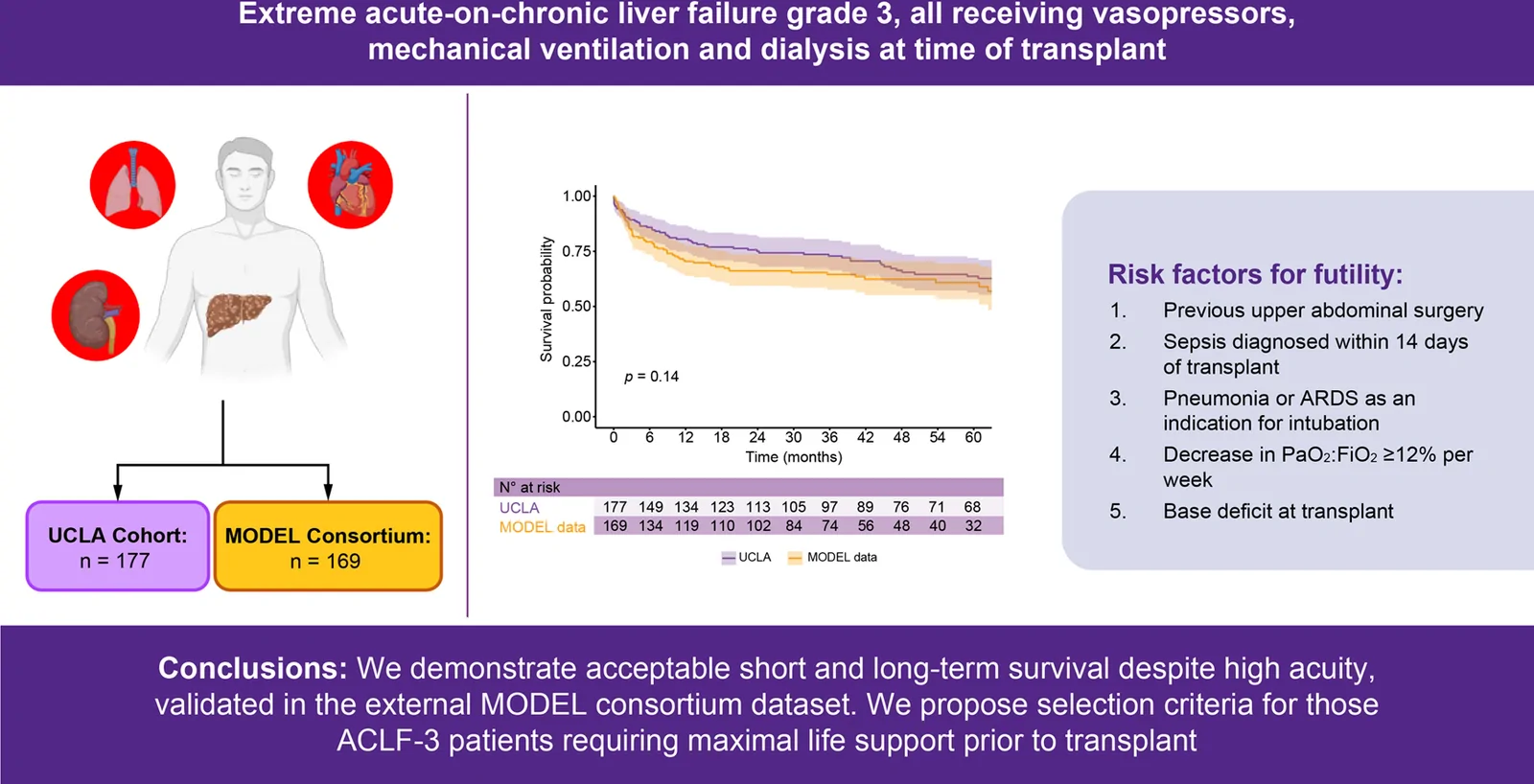

## Introduction

Acute-on-chronic liver failure (ACLF) is a syndrome characterized by acute hepatic decompensation, organ failure, and high short-term mortality.[1], [2], [3], [4], [5] Patients with cirrhosis who develop an acute decompensation are at risk for the development of ACLF with subsequent rapid organ dysfunction and an elevated mortality risk.[6], [7], [8] The European CANONIC trial (Consortium Acute-on-chronic Liver Failure in Cirrhosis) defined ACLF in three grades according to the number of associated organ failures.^1^^,^^9^ ACLF can be stratified using the Chronic Liver Failure Consortium (CLIF)-Organ failure score, which reflects the number and degree of organ failures and the CLIF-C ACLF score which predicts outcomes in patients with ACLF and may better assess risk of waitlist mortality in patients with ACLF than the model for end-stage liver disease (MELD) score.^10^ ACLF grade 3 (ACLF-3) is the most severe form of the disease where patients have at least three organ failures and is associated with 28-day waitlist mortality approaching 80%.^7^^,^[10], [11], [12], [13] In an analysis of the United Network for Organ Sharing (UNOS) database, ACLF-3 recipients had worse waitlist and post-transplant survival compared with status 1A recipients, traditionally considered to be the highest acuity liver transplant (LT) population.^12^

Because of the extremely high waitlist mortality of patients with ACLF-3, there is considerable interest in offering these patients early LT to afford them the greatest chance of survival.[14], [15], [16], [17], [18], [19], [20], [21] However, this is balanced against the concerns about poor post-transplant outcomes and stewardship of scarce donor livers. There have thus been considerable efforts to predict futility following transplant in ACLF-3 recipients and to identify risk factors for futile outcomes.^7^^,^[22], [23], [24], [25] In one large multicenter ACLF-3 study, five European centers contributed data on 152 ACLF-3 LT recipients and created the ‘transplantation for ACLF-3 model’ (TAM score) to predict 1-year post-transplant mortality.^24^ This study identified risk factors for 1-year post-transplant mortality including advanced age, lactate, respiratory failure, and increasing white blood cell count and were able to predict 1-year mortality with an AUROC of 0.88. Another recent study was reported by Hernaez *et al.*^25^ where 15 US centers contributed data on 735 patients with ACLF, 521 of whom were considered ‘severe’, including patients with ACLF 2 and 3. These data were used to create the novel Sundaram ACLF-LT-Mortality (SALT-M) score, which predicts 1-year post-transplant mortality in this group of patients with severe ACLF with a reported AUROC of 0.80.

Although the TAM and SALT-M scores represent significant contributions to the ability to discriminate patients with severe ACLF who may be at risk for 1-year post-transplant mortality, there is a spectrum of disease even within ACLF-3 that has not been specifically explored. The European definition of ACLF, based on the CLIF-C organ failure scoring system, assesses function of six organ systems (liver, kidney, brain, coagulation, circulation, and respiration).^10^ Based on this system, patients can be considered to have ACLF-3 with evidence of failure in any three of these organ systems. It is likely that a patient with acutely decompensated liver function, disordered coagulation, and hepatic encephalopathy would have a significantly different risk profile compared to a patient with respiratory failure requiring mechanical ventilation, circulatory failure requiring vasopressors, and renal failure requiring dialysis.^26^ In the original TAM score study, 73% of the cohort was mechanically ventilated, 70% on pressors, and 65% receiving dialysis at time of transplant. In the cohort used to create the SALT-M score, 48% were receiving mechanical ventilation, 52% were on vasopressors, and 84% were receiving dialysis at time of LT. We hypothesized that patients with ACLF-3 requiring concomitant mechanical ventilation, vasopressors, and dialysis – all forms of critical life support – represent a hyperacute subgroup of extreme ACLF-3 (eACLF-3) transplant recipients who have not specifically been analyzed in prior studies. We sought to characterize our single-center experience in transplanting these patients, with a specific goal of identifying risk factors for futility after transplant. Additionally, we sought to externally validate our findings using data from the Multi-Organ Dysfunction and Evaluation for Liver Transplantation (MODEL) Consortium, which was used to develop the SALT-M score.^25^

## Patients and methods

### Patient cohorts

We identified adult (age ≥18 years), first-time, non-status 1A, liver-only or liver–kidney transplant recipients at UCLA Medical Center from January 1, 2010 to December 31, 2021 through retrospective chart review. After exclusion, we identified patients with decompensation with one or more additional organ failures and stratified them based on ACLF score (1, 2, or 3). Within the patients with ACLF-3, we specifically identified recipients with acute decompensation of cirrhosis with multiple organ failures resulting in the need for mechanical ventilation, vasopressors, and renal replacement therapy (eACLF-3) at the time of transplantation. This study was approved by the UCLA institutional review board (IRB #11-001932).

Data collection for the MODEL Consortium has been previously described,^25^ approval from contributing authors was obtained and data had been collected under IRB approvals at each participating institution. From this cohort, we identified eACLF-3 LT recipients requiring vasopressors, on dialysis, and mechanically ventilated at time of LT.

### Data collection and definition of eACLF-3

Patients meeting the criteria for ACLF (grades 1–3) were identified and categorized at the time of LT based on the EASL-CLIF criteria. The patients with severe ACLF-3 were then further stratified into eACLF-3, which included only those who were mechanically ventilated, receiving vasopressors, and receiving renal replacement therapy at the time of LT. If patients were extubated, weaned off pressors, or weaned off dialysis before LT, they were not included as part of this eACLF-3 cohort. For the eACLF-3 cohort, pre-transplant clinical characteristics were collected at time of intensive care unit (ICU) admission and at time of transplant. Several variables were calculated using the change in value from ICU admission to transplant, including change in MELD score and change in the ratio of the partial pressure of oxygen in arterial blood to the fraction of inspiratory oxygen concentration (PaO_2_:FiO_2_ ratio). The indication for mechanical ventilation was recorded as either strictly for airway protection or due to pulmonary pathology including pneumonia and acute respiratory distress syndrome (ARDS). The indication for ventilation was determined through manual chart review with physician review of chest X-rays, X-ray radiology reports, and any relevant microbiological data including sputum and blood cultures. Sepsis was considered if patients had clinical suspicion for sepsis with an identified (culture proven) infectious source (pneumonia, spontaneous bacterial peritonitis, urinary tract infection, or other source). We included a sepsis duration variable that signified if a patient was diagnosed with sepsis longer than 2 weeks before transplant, within 2 weeks, or not diagnosed with sepsis.

### Outcomes

The primary outcome of interest was futility following transplant, defined as 1-year post-transplant all-cause mortality or in-hospital mortality. We included in-hospital mortality in the definition to capture patients who receive prolonged intensive care and life support following transplant, ultimately experiencing mortality in the hospital, which should be considered a futile outcome even if it did not occur within 1 year. Extensive secondary outcomes were evaluated and reported, including 5-year overall survival, post-LT ICU and total length of hospitalization, total time of intubation, number of reoperations, planned *vs*. unplanned reoperations, time to reoperation, postoperative respiratory failure (requiring re-intubation), neurological failure (ischemic stroke or cerebral hemorrhage), cardiac failure (heart failure leading to low output syndrome), sepsis, portal vein thrombosis, hepatic artery thrombosis, primary graft non-function, discharge to skilled nursing facility, discharge to long-term acute care hospital, readmission within 1 month after discharge, dialysis dependence following transplant, tracheostomy-dependence, graft failure requiring re-transplant, and cause of death.

### Statistical analysis

Analyses were conducted using SAS (SAS Institute, Cary, NC, USA) and R version 4.1.0 (R Foundation for Statistical Computing, Vienna, Austria). Any *p* values <0.05 were considered significant.

Baseline characteristics at time of ICU admission and at LT were compared between the futile and non-futile groups. Characteristics were also compared between patients who were eACLF-3 and non-eACLF-3 at our center as well as patients with eACLF-3 at our center and in the MODEL Consortium. Group medians were displayed with IQR ranges and compared using the Mann–Whitney *U* test. Categorical variables were displayed as number (%) and compared using the Χ^2^ test. We compared survivals between the patients with futile and non-futile eACLF-3 as well as the patients with eACLF-3 at our center and in the MODEL Consortium using the Kaplan–Meier method. Although our focus was on patients with eACLF-3, we also compared overall survival between patients with ACLF 1, 2, and 3 using the Kaplan–Meier method. Survival differences were assessed for statistical significance using the log-rank test in all comparisons.

Univariate logistic regression analysis with futility modeled as the outcome was conducted for all collected variables. For features that were calculated based on change in values from ICU admission to LT, we rigorously tested different binarized threshold value cut-offs and determined quantities of change most predictive of futility empirically. Features for the final multivariate logistic regression model were selected using stepwise selection based on the Akaike information criterion (AIC) and clinical relevance. Model performance was assessed using fivefold cross-validation on the internal UCLA data with each training-validation split stratified on the futility outcome. A decision threshold was selected at the lowest predicted probability resulting in a positive predictive value (PPV) of ≥50% in each training fold. Using each training probability threshold, sensitivity, specificity, and PPV were calculated on each validation fold. AUROC was also calculated across the five folds and all evaluation metrics were reported with mean and standard deviation. Model calibration was assessed using the Brier score and the Hosmer–Lemeshow test. A risk score was created by dividing each feature coefficient by the smallest coefficient value and rounding to the nearest integer. The median predicted probability used as a decision threshold in each training fold was used to determine the risk score threshold over which futility should be strongly considered. Missing values for variables in the final multivariate model were imputed using multiple imputation by chained equations using the ‘MICE’ R package (R Foundation for Statistical Computing).^27^

#### Removal of simultaneous liver–kidney transplant recipients

Patients receiving simultaneous liver–kidney transplant were removed in this sensitivity analysis. Post-transplant survival was plotted using the Kaplan–Meier method and a multivariate logistic regression model was created and evaluated using the same procedures as above.

## Results

**Fig. 1:**
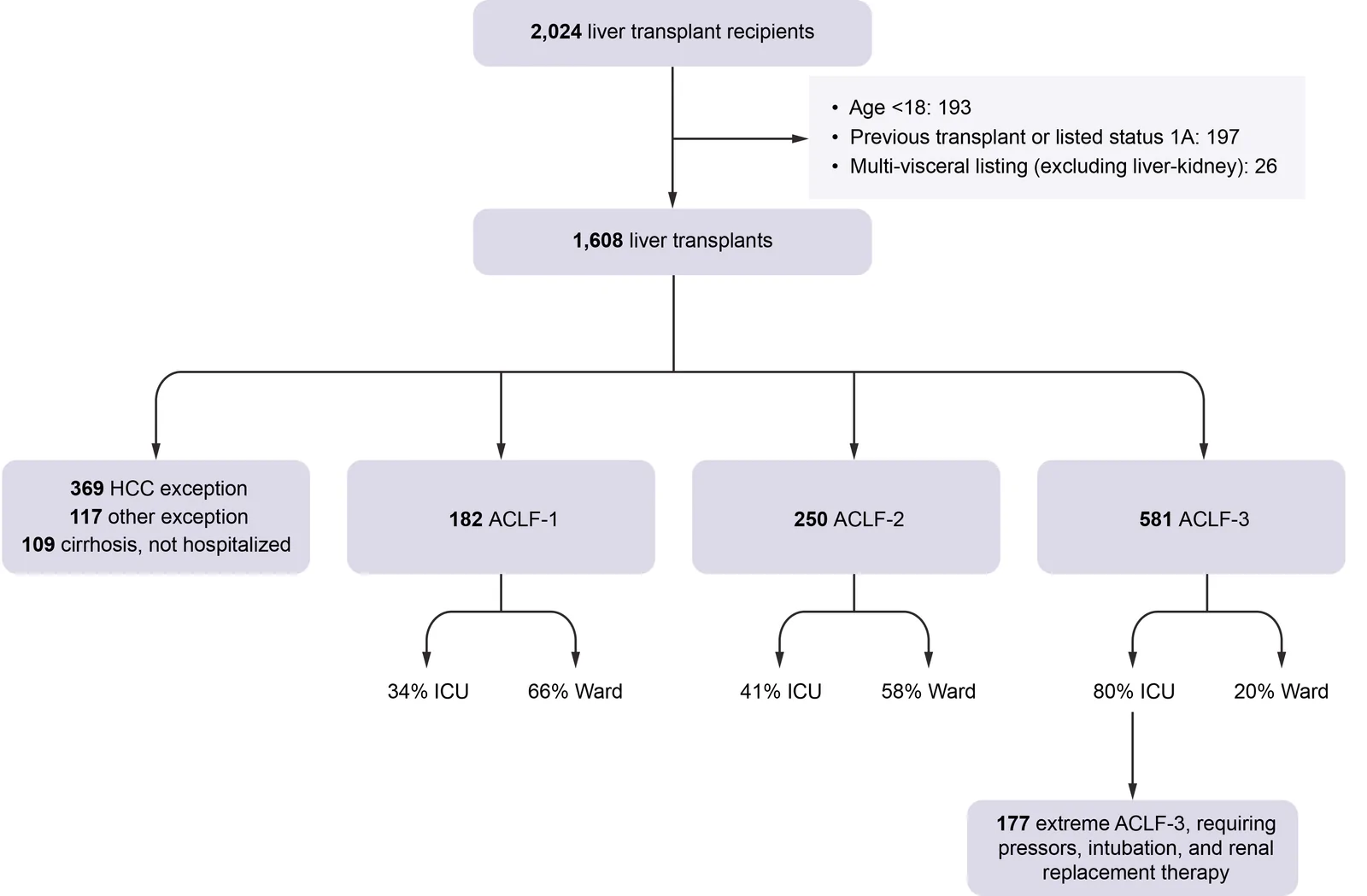
Selection of patient cohorts, with exclusions made for pediatric patients, those with previous transplants, status 1A recipients, multi-visceral recipients (excluding liver–kidney), and transplants outside the study timeframe. The extreme ACLF-3 cohort was identified from among the patients with ACLF-3 who were transplanted.

Of 2,024 total LTs performed at our center, 1,608 met study inclusion criteria, of which 182 (11%) were ACLF-1, 250 (16%) were ACLF-2, and 581 (36%) were for ACLF-3 (Fig. 1). The remaining 595 (37%) patients were those with hepatocellular carcinoma (369, 23%), non-HCC related MELD exception points (117, 7%), or decompensated cirrhosis not requiring hospitalization before transplant (109, 7%). Of the 581 patients with ACLF-3, 177 (30%) represented patients with eACLF-3 all requiring mechanical ventilation, vasopressors, and receiving renal replacement therapy at time of LT. Baseline demographic comparisons between patients with non-extreme ACLF-3 and eACLF-3 are shown in Table S1. Annual transplant center volume, number (%) of transplants for ACLF-3, and transplant center and donor service area median MELD at transplant during successive years of the study period are shown in Table S2. There were 35 patients with eACLF-3 who experienced the futile outcome (20%), with baseline characteristics shown in Table 1. Compared with patients with non-futile eACLF-3, those with futile eACLF-3 were significantly more likely to have hepatitis C (43% *vs*. 21%, *p* = 0.012), previous upper abdominal surgery (13% *vs.* 11%, *p* = 0.042), had a greater base deficit at time of transplant (median, IQR: 0.0, −2.0–1.0 *vs*. 1.0, −1.0–2.0, *p* = 0.004), were more likely to be intubated at LT for pneumonia or ARDS (77% *vs.* 54%, *p* = 0.011), and were more likely to have sepsis within 14 days before transplant (77% *vs.* 54%, *p* = 0.040). The two groups did not significantly differ in MELD or CLIF-C ACLF scores at ICU admission or at transplant, and had similar waitlist times, duration of pre-transplant total and ICU admission, and similar proportion of outside hospital admission and duration of admission before transferring care to our center. Regarding donor and operative characteristics, patients with futile eACLF-3 received liver allografts with greater median donor risk index (median, IQR: 1.4, 1.2–2.3 *vs.* 1.3, 1.1–1.6, *p* = 0.011), received more packed red blood cell transfusions during transplant (median, IQR: 31.0 (18.0–50.0) *vs.* 23.0 (15.0–36.0), *p* = 0.049), and were more likely to have required venovenous bypass during transplant (86% *vs.* 65%, *p* = 0.017) compared with the non-futile eACLF-3 cohort.

**Table 1:** Baseline demographic comparisons between patients with ACLF grade 3 with and without the futile outcome.

Variable	Total (N = 177)	Non-futile (n = 142)	Futile (n = 35)	*p* values
**Demographic**				
Age (y)	58.0 (49.6–64.0)	57.6 (51.9–63.3)	58.3 (49.1–64.1)	0.70
Sex = M	89 (50)	72 (51)	17 (49)	0.82
BMI (kg/m^2^)	26.6 (23.6–31.7)	26.5 (23.4–31.8)	27.8 (24.1–31.4)	0.79
Primary diagnosis				0.012
Alcohol	65 (37)	54 (38)	11 (31)	
MASLD	45 (25)	38 (27)	7 (20)	
Hepatitis C	34 (19)	20 (14)	14 (40)	
Autoimmune/cholestasis	21 (12)	19 (14)	2 (6)	
Other	12 (7)	10 (7)	2 (6)	
Prior abdominal surgery	70 (40)	50 (35)	20 (57)	0.018
Type of abdominal surgery				0.042
Previous upper abdominal surgery	23 (13)	15 (11)	8 (23)	
Previous lower abdominal surgery	33 (19)	26 (19)	7 (20)	
Previous upper and lower abdominal surgery	13 (7)	8 (6)	5 (14)	
Diabetes mellitus	54 (31)	46 (32)	8 (23)	0.27
Hypertension	54 (31)	46 (32)	8 (23)	0.27
Hyperlipidemia	32 (18)	29 (20)	3 (9)	0.10
Metabolic syndrome	39 (22)	36 (25)	3 (9)	0.032
Coronary artery disease	26 (15)	20 (14)	6 (17)	0.65
Cardiac arrythmia	38 (21)	27 (19)	11 (31)	0.11

**Pre-LT acuity**				
MELD-Na on ICU admission	38.4 (35.4–41.5)	38.9 (35.9–41.5)	37.8 (33.6–41.9)	0.26
CLIF-C ACLF on ICU admission	58.5 (54.0–66.8)	58.4 (53.6–64.1)	58.2 (52.4–65.9)	0.79
Number of organ failures at ICU	3.0 (1.0–4.0)	2.0 (2.0–3.0)	3.0 (2.0–4.0)	0.20
CLIF-C organ failure score at ICU	13.0 (10.0–15.0)	13.0 (12.0–14.0)	13.0 (12.0–15.0)	0.38
MELD-Na at LT	41.0 (38.5–42.8)	41.0 (38.3–43.0)	40.7 (39.1–42.8)	0.90
CLIF-C ACLF at LT	68.0 (62.8–73.0)	67.8 (62.8–71.9)	68.7 (65.7–72.5)	0.34
Number of organ failures at LT	3.0 (3.0–4.0)	4.0 (4.0–5.0)	4.0 (4.0–5.0)	0.48
CLIF-C organ failure score at LT	15.0 (14.0–16.0)	16.0 (14.0–17.0)	16.0 (15.0–17.0)	0.29
Base excess at LT	0.0 (-1.0–2.0)	1.0 (-1.0–2.0)	0.0 (-2.0–1.0)	0.004

**Reason for mechanical ventilation**				0.011
Lung pathology (pneumonia/ARDS)	103 (58)	76 (54)	27 (77)	
Hepatic encephalopathy/airway protection	74 (42)	66 (47)	8 (23)	

**Pre-LT sepsis characteristics**				
Sepsis within 1 month before LT	105 (59)	84 (60)	21 (60)	0.96
Interval from sepsis to LT				0.040
No infection	39 (22)	35 (25)	4 (11)	
≤14 days	103 (58)	76 (54)	27 (77)	
>14 days	35 (20)	31 (22)	4 (11)	
Infection with MRDO	17 (10)	12 (9)	5 (14)	0.18
Source of sepsis				
Pneumonia	96 (54)	74 (52)	22 (63)	0.38
SBP	3 (2)	3 (2)	0 (0)	–
UTI	44 (25)	32 (23)	12 (34)	0.24
Other	12 (7)	7 (5)	5 (14)	0.12
Infection with MRDO	17 (10)	12 (9)	5 (14)	0.18

**Pre-LT hospitalization characteristics**				
Listing date to LT (d)	18.0 (6.0–157.0)	18.0 (6.0–146)	31.0 (7.0–228)	0.54
Duration of preoperative admission (d)	21.0 (14.0–31.0)	21.0 (15.0–32.0)	17.0 (11.0–29.0)	0.21
Duration of preoperative ICU admission (d)	17.0 (9.0–23.0)	17.0 (9.0–23.0)	13.0 (9.0–24.0)	0.74
Duration of preoperative RRT (d)	12.0 (6.0–20.0)	13.0 (7.0–21.0)	9.0 (5.0–12.5)	0.008
ICU admission before listing	91 (51)	74 (52)	17 (49)	0.85
OSH admission	131 (74)	108 (76)	23 (66)	0.21
Duration of OSH admission (d)	4.0 (0.0–11.0)	5.0 (0.0–12.0)	0.0 (0.0–5.0)	0.07

**Donor and operative characteristics**				
Donor risk index	1.3 (1.1–1.6)	1.3 (1.1–1.6)	1.4 (1.2–2.3)	0.011
DCD donor	4 (2)	3 (2)	1 (9)	1.00
Cold ischemic time (h)	7.0 (6.0–8.0)	7.2 (6.2–8.3)	7.2 (6.0–8.2)	0.68
Simultaneous liver–kidney transplant	14 (8)	14 (10)	0 (0)	–
PRBC transfusion (u)	23.0 (16.0–36.0)	23.0 (15.0–36.0)	31.0 (18.0–50.0)	0.049
Venovenous bypass	122 (69)	92 (65)	30 (86)	0.017
Worst base excess	−8.7 (−11.9 to −6.5)	−8.3 (−11.0 to −6.0)	−9.4 (−13.1 to −7.0)	0.033
Base excess at end of LT	−2.0 (−4.0–1.1)	−1.3 (−3.6–1.5)	−3.6 (−5.4–0.0)	0.013

Categorical variables are displayed as n (%) and differences were assessed using the Χ^2^ test (significant when *p* <0.05). Continuous variables are displayed as median (IQR) and differences were assessed using the Mann–Whitney *U* test (significant when *p* <0.05). ACLF, acute-on-chronic liver failure; ARDS, acute respiratory distress syndrome; CLIF-C, Chronic Liver Failure Consortium; DCD, donation after circulatory death; ICU, intensive care unit; LT, liver transplant; MELD, model for end-stage liver disease; MRDO, multi-drug resistant organism; OSH, outside hospital; PRBC, packed red blood cells; RRT, renal replacement therapy; SBP, spontaneous bacterial peritonitis; UTI, urinary tract infection.

**Fig. 2:**
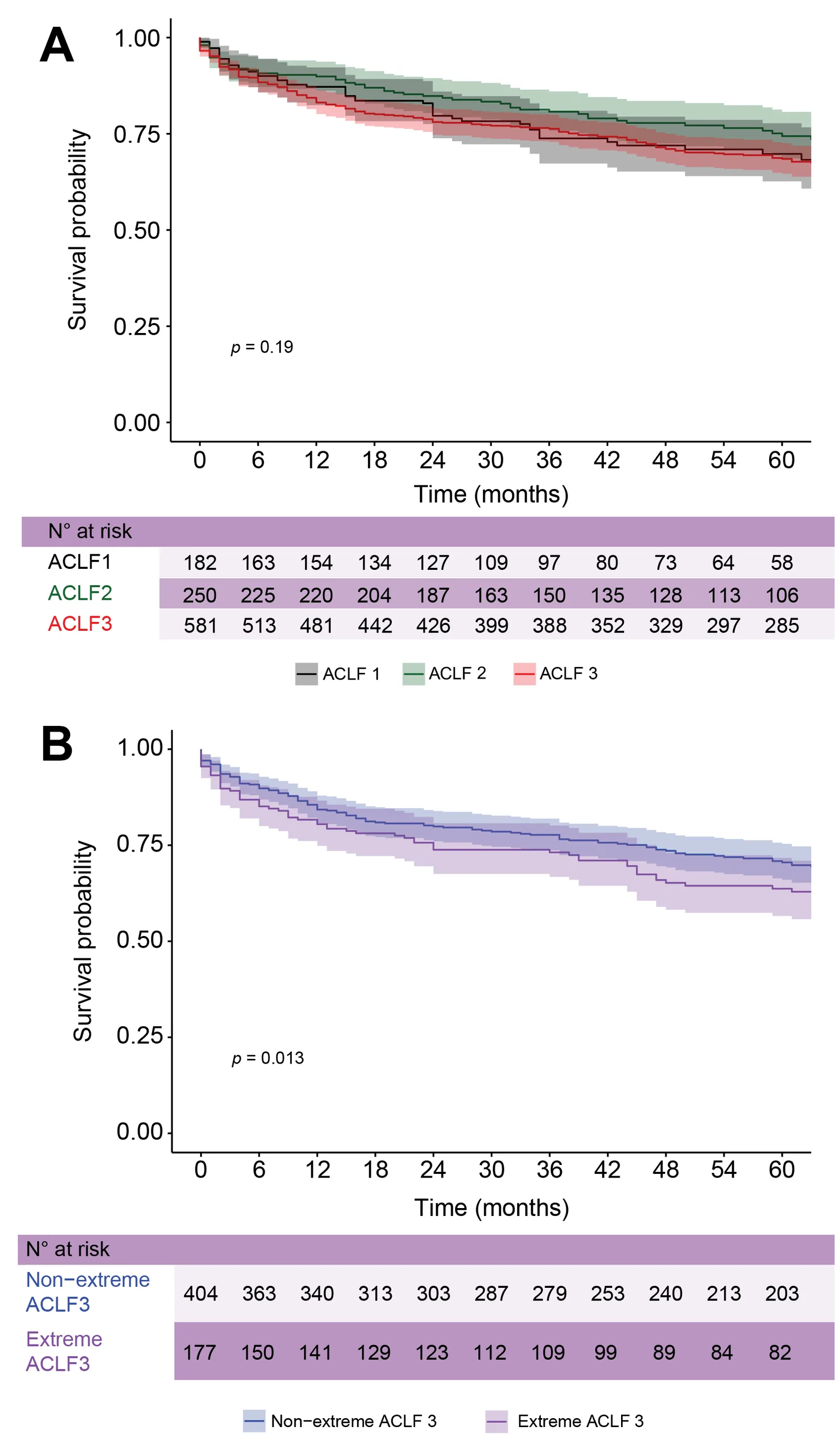
Survival comparisons between patients with ACLF 1, 2, and 3, and non-extreme and extreme ACLF 3. (A) Survival comparisons between patients with ACLF 1, 2, and 3 (Kaplan–Meier method, log-rank test: *p* = 0.19). (B) Survival comparison between patients with non-extreme ACLF 3 and extreme ACLF 3 (Kaplan–Meier method, log-rank test: *p* = 0.013, statistically significant). Shaded area represents 95% confidence intervals.

Overall survival was decreased in patients with ACLF-3 compared with those with ACLF-1 and ACLF-2 (though not statistically significantly), with survivals of 87%, 90%, and 83% at 1-year, 74%, 81%, and 76% at 3-years, and 70%, 74%, and 69% at 5 years for patients with ACLF-1, ACLF-2, and ACLF-3, respectively (Fig. 2A, overall *p* = 0.19). In a sub-analysis of patients with ACLF-3, patients with eACFL-3 had lower 1-, 3-, and 5-year survival compared with non-eACLF-3 (81%, 73%, and 63% *vs.* 84%, 77%, and 71%, respectively; Fig. 2B, *p* = 0.013).

Details of the causes of death and comparisons of secondary outcomes between the eACLF-3 non-futile and futile groups are shown in Table 2. Patients in the futile group had a longer median time of post-LT intubation (45.0 days, IQR: 7.0–94.0 *vs.* 8.0 days, IQR: 3.0–27.5, *p* = 0.003), and a larger proportion of patients with two or greater unplanned reoperations (20% *vs*. 11%, *p* = 0.025). Additionally, patients in the futile group were more likely to have had postoperative respiratory, neurological, and cardiac failure (97%, 60%, 69% *vs.* 59%, 9%, 10%, *p* <0.001 for all comparisons). In the futile group, the most common cause of death within 1 year was sepsis with multisystem organ failure (23%).

**Table 2:** Secondary outcomes in patients with extreme ACLF-3 with comparisons made between the non-futile and futile groups and specific causes of death in the futile group.

Variable	Total (N = 177)	Non-futile (n = 142)	Futile (n = 35)	*p* values
Post-LT ICU length of stay (days)	29.0 (14.0–54.0)	26.0 (15.0–49.0)	44.0 (10.0–73.0)	0.41
Post-LT length of stay (days)	53.0 (32.0–78.5)	53.0 (33.0–80.0)	56.0 (14.0–80.0)	0.65
Time of intubation post-LT	9.0 (3.0–44.0)	8.0 (3.0–27.5)	45.0 (7.0–94.0)	0.003
Number of reoperations				0.071
0	76 (43)	64 (45)	12 (34)	
1	61 (35)	51 (36)	10 (29)	
≥2	40 (23)	27 (19)	13 (37)	
Unplanned reoperations				0.025
0	120 (68)	103 (73)	17 (49)	
1	34 (19)	23 (16)	11 (31.)	
≥2	23 (13)	16 (11)	7 (20)	
Time to first reoperation		8.0 (4.0–16.0)	5.0 (2.0–12.0)	0.25
Postoperative respiratory failure	118 (67)	84 (59)	34 (97)	<0.001
Postoperative neurological failure	33 (19)	12 (9)	21 (60)	<0.001
Postoperative cardiac failure	38 (22)	14 (10)	24 (69)	<0.001
Postoperative sepsis	95 (54)	76 (54)	19 (54)	0.013
Portal vein thrombosis	2 (1)	1 (1)	1 (3)	0.28
Hepatic artery thrombosis	9 (5)	5 (4)	4 (11)	0.057
Primary graft non-function	6 (3)	3 (2)	3 (9)	0.171
Discharge to SNF	10 (6)	8 (6)	2 (6)	<0.001
Discharge to LTACH	11 (6)	11 (8)	0 (0)	<0.001
Post-LT readmission within 1 month	43 (24)	39 (28)	4 (11)	0.046
Post-LT dialysis dependence	22 (12)	16 (11)	6 (17)	0.35
Post-LT tracheostomy	47 (27)	34 (24)	13 (37)	0.11
Re-LT	10 (6)	8 (6)	2 (6)	0.57
Discharged following index LT	–	–	9 (26)	–
Cause of death				
Cerebral hemorrhage	–	–	3 (9)	–
HAT	–	–	2 (6)	–
MSOF – no sepsis	–	–	3 (9)	–
PE	–	–	1 (3)	–
Pneumonia with sepsis, MSOF	–	–	3 (9)	–
PNF	–	–	3 (9)	–
Delayed graft function with MSOF			3 (9)	
Sepsis with MSOF	–	–	8 (23)	–
Other			6 (17)	
Unknown	–	–	3 (9)	–

Categorical variables are displayed as n (%) and differences were assessed using the Χ^2^ test (significant when *p* <0.05). Continuous variables are displayed as median (IQR) and differences were assessed using the Mann–Whitney *U* test (significant when *p* <0.05). ACLF, acute-on-chronic liver failure; HAT, hepatic artery thrombosis; ICU, intensive care unit; LT, liver transplant; LTACH, long-term acute care hospital; MSOF, multisystem organ failure; PE, pulmonary embolism; PNF, primary non-function; SNF, skilled nursing facility.

Univariate modeling results evaluating all candidate factors associated with the futile outcome are detailed in Table S3 (categorical variables) and Table S4 (continuous variables). Tested thresholds for continuous variables converted to categorical factors are shown in Table S5. The final selected multivariate logistic regression model from stepwise selection had an AIC of 158 with complete model results is shown in Table 3. The final selected model features for post-LT futility included previous upper abdominal surgery (odds ratio [OR] = 4.6; 95% CI, 1.8–12.1, *p* = 0.002), sepsis within 14 days before LT (OR = 5.9; 95% CI, 1.5–24.1, *p* = 0.013), requiring ventilatory support as a result of pneumonia or ARDS (OR = 3.5; 95% CI, 1.4–9.2, *p* = 0.010), worsening PaO_2_:FiO_2_ ≥12% per week (OR = 3.9; 95% CI, 1.4–10.6, *p* = 0.008), and increasing pre-LT base deficit (OR = 1.27; 95% CI, 1.1–1.5; *p* = 0.007). The Brier score for the final model was 0.12 and Hosmer–Lemeshow *p* value was 0.38 (≥0.05 suggests no significant differences between observed and predicted values). On fivefold cross-validation, the final model predicted futility in patients with eACLF-3 with a mean sensitivity of 0.57 (SD 0.23), specificity of 0.81 (SD 0.11), PPV of 0.45 (SD 0.17), and AUROC of 0.74 (SD 0.15). The median decision threshold used was a predicted probability of 0.25. The reported risk score can be calculated using values in Table 3 and the identified risk score threshold was 8, corresponding to a predicted probability of 0.25, above which futility occurred in ∼50% of patients.

**Table 3:** Results of multivariate logistic regression modeling futility.

Variable	Risk score points	β-coefficients	OR	95% CI	*p* values
Intercept	0	−4.0			
Previous upper abdominal surgery	6	1.5	4.6	1.8–12.1	0.002
Sepsis duration (ICU to LT)					
None	0	–	–	–	–
≤14 days	4	1.8	5.9	1.5–24.1	0.013
>14 days	1	1.0	2.6	0.7–9.6	0.14
Intubation indication					
Airway protection	0	–	–	–	–
Pneumonia/ARDS	6	1.3	3.5	1.4–9.2	0.010
Decrease in PaO_2_:FiO_2_ ≥12% per week from ICU to LT	7	1.4	3.9	1.4–10.6	0.008
Base deficit at LT	5	0.2	1.3	1.1–1.5	0.007

Displayed *p* values are determined using the Wald test, *p* <0.05 was considered significant. ARDS, acute respiratory distress syndrome; ICU, intensive care unit; LT, liver transplant; OR, odds ratio.

### Removal of simultaneous liver–kidney transplant recipients

Overall post-transplant survival was similar when simultaneous liver–kidney transplant recipients were removed from analysis with 1-, 3-, and 5-year survival of 78%, 71%, and 61%, respectively; Fig. S1). Overall, the logistic regression modeling efforts showed no significant differences when simultaneous liver–kidney transplant recipients were excluded. The same five features were selected for the final model in stepwise regression. The median selected decision threshold was at a predicted probability of 0.26. The average AUROC was 0.77 (SD 0.13), sensitivity 0.66 (SD 0.13), specificity 0.77 (0.12), and PPV 0.48 (SD 0.22).

### Comparison to external MODEL Consortium cohort

**Fig. 3:**
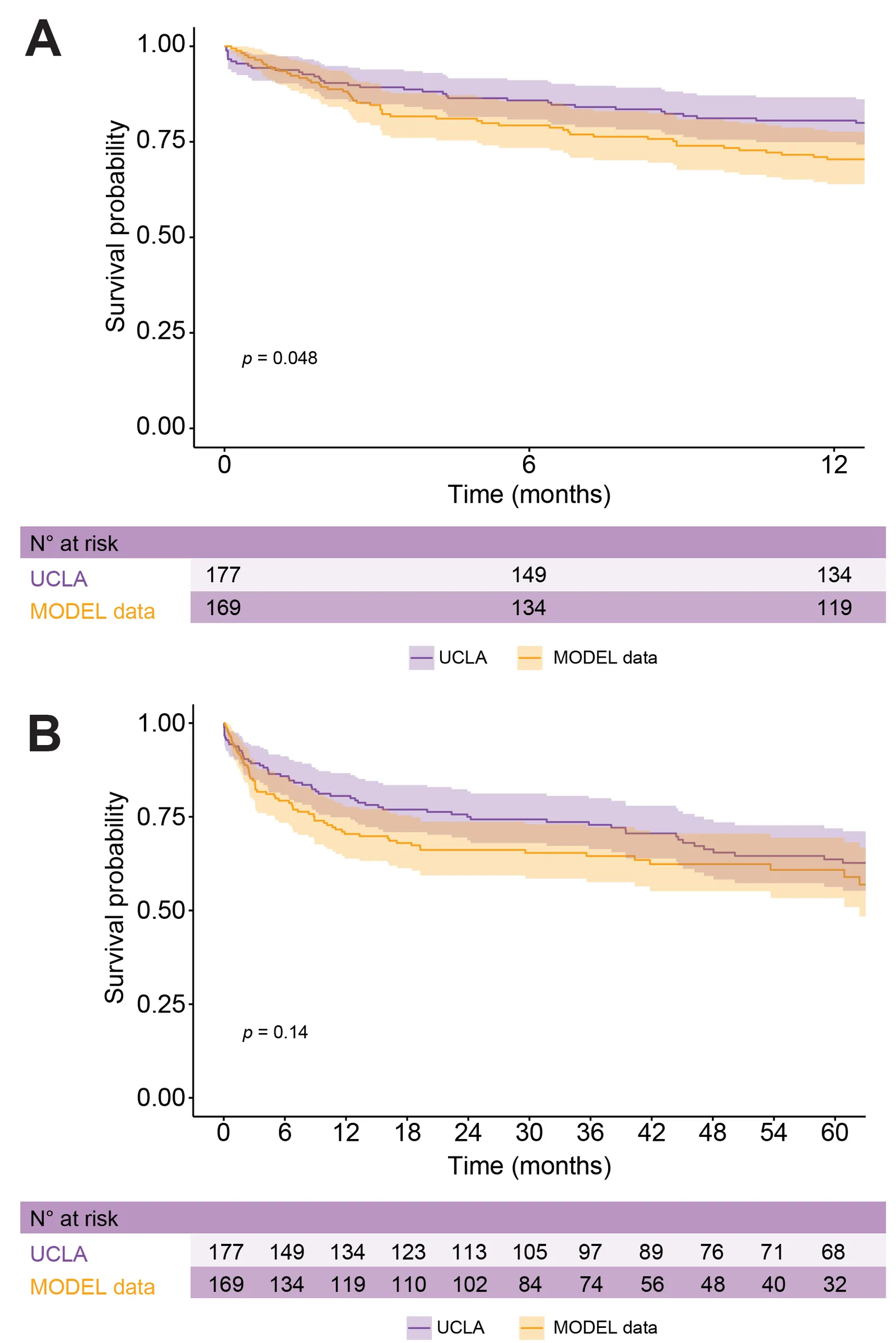
One-year and overall survival comparisons between patients with extreme ACLF-3 at UCLA (purple) and in the MODEL Consortium (orange). Survival differences were assessed using the Kaplan–Meier method and log-rank test (A: *p* = 0.048, statistically significant; B: *p* = 0.14). Shaded area represents 95% confidence intervals.

There were 169 patients meeting criteria for eACLF-3 in the MODEL Consortium dataset. Complete comparisons of demographics and outcomes are shown in Table 4. In terms of outcomes, MODEL patients had shorter median post-LT ICU lengths of stay (9 days *vs.* 29 days, *p* <0.001), shorter median total lengths of stay (22 days *vs.* 53 days, *p* <0.001), shorter median duration of post-LT intubation (2 days *vs.* 9 days, *p* <0.001), fewer re-transplants (1% *vs.* 6%, *p* = 0.017) compared to the UCLA cohort. However, MODEL eACFL-3 patients experienced more readmissions 1 month after LT (36% *vs.* 24%, *p* = 0.030), with a larger proportion of patients experiencing the futile outcome (30% *vs.* 20%, *p* = 0.046). MODEL cohort patients had worse 3-, 6-, and 12-month survivals of 83%, 79%, and 70%, compared with the UCLA cohort with 89%, 85%, and 80%, respectively (Fig. 3A, *p* = 0.048). However, overall survival was equivalent, with the MODEL cohort patients having 1-year, 3-year, and 5-year overall survivals of 70%, 65%, and 59%, compared with 80%, 73%, and 63%, respectively, in the UCLA cohort (Fig. 3B, *p* = 0.14).

**Table 4:** Baseline demographic and outcomes comparison in patients with extreme ACLF-3 at UCLA and external MODEL Consortium cohort.

Variable	UCLA (n = 177)	MODEL (n = 169)	*p* values
**Demographic**			
Age (years)	58.00 [49.6, 63.9]	55.00 [46.0, 61.3]	0.016
Sex = M	89 (50)	78 (46.2)	0.51
BMI (kg/m^2^)	26.6 [23.6, 31.7]	29.9 [24.9, 36.0]	0.001
Primary diagnosis			0.39
Alcohol	65 (37)	65 (39)	
MASLD	45 (25)	27 (16)	
Hepatitis C	34 (19)	29 (17)	
Autoimmune/cholestasis	21 (12)	25 (15)	
Other	12 (7)	23 (14)	
Prior abdominal surgery	70 (40)	70 (42)	0.77
Diabetes mellitus	54 (31)	40 (24)	0.20

**Pre-LT acuity/hospitalization characteristics**			
MELD-Na on ICU admission	38.4 [35.4, 41.5]	36.0 [32.0, 40.0]	<0.001
MELD-Na at LT	41.0 [38.5, 42.8]	37.7 [34.8, 41.3]	<0.001
Reason for mechanical ventilation			0.009
Lung pathology (pneumonia/ARDS)	103 (58)	71 (43)	
Hepatic encephalopathy/airway protection	74 (42)	98 (58)	
PaO_2_:FiO_2_ ratio at ICU admission	247.1 [165.0, 334.2]	255.0 [161.6, 349.3]	0.92
PaO_2_:FiO_2_ ratio at LT	303.3 [230.0, 387.5]	259.0 [211.4, 330.6]	0.003
Preoperative sepsis	105 (60)	96 (57)	0.66
Listing date to LT (days)	18.0 [6.0, 157.0]	7.0 [3.0, 43.5]	<0.001
Duration of preoperative ICU admission (days)	17.0 [9.0, 23.0]	9.0 [5.0, 16.0]	<0.001

**Donor and operative characteristics**			
Donor age (years)	34.0 [22.0, 49.0]	35.5 [26.0, 49.8]	0.14
Cold ischemic time (h)	7.0 [6.0, 8.0]	5.9 [4.4, 7.9]	0.001
DCD	4 (2)	18 (11)	0.003

**Outcomes**			
Post-LT ICU length of stay (days)	29.0 [14.0, 54.0]	9.0 [4.0, 18.0]	<0.001
Post-LT length of stay (days)	53.0 [32.0, 78.5]	22.0 [14.0, 36.0]	<0.001
Post-LT duration of intubation	9.0 [3.0, 44.0]	2.0 [1.0, 5.3]	<0.001
Extubated after LT	20 (11)	7 (4)	0.25
PNF	6 (3)	5 (3)	0.81
HAT	9 (5)	2 (1)	0.08
Discharge location			0.007
Home	76 (53)	92 (63)	
LTAC	11 (6)	20 (14)	
Rehab or SNF	57 (40)	35 (24)	
Post-LT readmission within 1 month	43 (24)	60 (36)	0.030
Biliary complications	29 (17)	30 (18)	0.84
Re-LT	10 (6)	1 (1)	0.017
Futility	35 (20)	50 (30)	0.046

Categorical variables are displayed as n (%) and differences were assessed using the Χ^2^ test (significant when *p* <0.05). Continuous variables are displayed as median (IQR) and differences were assessed using the Mann–Whitney *U* test (significant when *p* <0.05). ACLF, acute-on-chronic liver failure; ARDS, acute respiratory distress syndrome; CLIF-C, Chronic Liver Failure Consortium; DCD, donation after circulatory death; ICU, intensive care unit; LT, liver transplant; MASLD, metabolic dysfunction-associated steatotic liver disease; MELD, model for end-stage liver disease; MODEL, Multi-Organ Dysfunction and Evaluation for Liver Transplantation; OSH, outside hospital; SNF, skilled nursing facility.

## Discussion

In this study, we report the largest single-center experience of ACLF-3 (n = 581) and the only study to define a unique subset of patients with eACLF-3 receiving maximal life support (mechanical ventilation, vasopressors, and renal replacement therapy) at time of LT. Twenty percent of our eACLF-3 cohort experienced a futile outcome, making the overall 5-year survival 63%, which may be considered an unacceptably high short-term mortality to justify the broad use of scarce donor livers. However, considering the high acuity of these patients with eACLF-3, it was remarkable to see that patients with ACLF-3 on maximal life support can achieve acceptable long-term survival with appropriate selection.^28^ The 177 patients with eACLF-3 examined at our center had overall consistent patient characteristics and outcomes when compared with the 169 patients in the MODEL Consortium external validation cohort.

As it pertains to this unique population of patients with eACLF-3, significant risk factors for futility in this study included a decrease in PaO_2_:FiO_2_ ratio, sepsis diagnosed within 14 days of LT, and the indication for mechanical ventilation. In a 2021 expert consensus panel on futility in LT for patients with ACLF-3, Weiss *et al.*^21^ reported their prescribed criteria for futility in ACLF-3 including persistent fever or <72 h of antimicrobial therapy in the case of ongoing sepsis, PaO_2_:FiO_2_ ratio <150 at LT, levophed dose >1 μg/kg per min, serum lactate >9 mmol/L. Based on our data, the receipt of antimicrobial therapy for 72 h before LT in the case of sepsis may be inadequate for patients with eACLF-3 on maximal life support, with a longer observation period between sepsis onset and LT allowing for optimal treatment of sepsis and affording the greatest reduction in risk of futility. The decision to proceed with LT in a patient with sepsis must balance the concern about delaying transplant too long, resulting in further decompensation and an inability to perform surgery safely with the risk of infection recrudescence following LT with the addition of immunosuppression. Additionally, the incorporation of acuity measure changes from ICU admission to LT may be better suited to this population with a decrease in PaO_2_:FiO_2_ ratio from ICU to LT being more predictive of futility than a specific value cut-off at LT. Given that ACLF is associated with a rapid decompensation leading to organ failure, the rate of change of decompensation may better inform prognosis and the likelihood of futility following LT than acuity measures at LT alone.^29^^,^^30^

To our knowledge, this is the first study that specifically analyzed the indication for mechanical ventilation in patients with ACLF-3 as a predictor of post-LT outcomes. Determining indications for intubation can be a difficult task, requiring manual physician chart review with analysis of chest X-rays and culture data. Neither the TAM score nor the SALT-M score included indication for ventilation as a risk factor in the final model and these data are not available in national transplant registries, highlighting the significance of the elucidation of this specific risk factor in this study. Patients with ACLF-3 can require mechanical ventilation for a variety of reasons, and it is not surprising that those intubated for airway protection as a result of severe hepatic encephalopathy or esophageal variceal bleeding with no underlying pulmonary pathology have a lower risk of futility than those intubated for respiratory failure because of pneumonia or ARDS. In fact, among the 35 patients who experienced futility, 29 (83%) of them were intubated for pulmonary pathology preoperatively, with 21 (60%) patients never extubated postoperatively and expiring while intubated. In those futile patients who were extubated postoperatively, eight (23%) were re-intubated before discharge or mortality.

In addition to indication for mechanical ventilation, worsening base deficit at LT was identified as a significant risk factor for futility, which is consistent with prior reports of increasing lactate level (another marker of tissue ischemia and systemic inflammation) being associated with futility in patients with ACLF-3.^21^^,^^24^ Lactate was not routinely collected at all time points at our center so we could not assess its impact on futility. Given that nearly 35% of the futile patients had multisystem organ failure as a cause of death, base deficit may be an important indicator of unrecoverable global tissue ischemia that portends futile outcomes even with LT. Additionally, within the 35% who died as a result of multisystem organ failure, 29% had associated sepsis, again signifying the prognostic importance of sepsis in this population. Shorter duration of sepsis with worsening base deficit may suggest overwhelming sepsis leading to multisystem organ failure that cannot be salvaged even with LT.

An increasing donor risk index was correlated with futility in demographic comparisons, validating that high-risk donor livers are ill suited to such critically ill patients with ACLF-3 on maximal life support.^31^^,^^32^ In fact, in our cohort only four patients with eACLF-3 received donation after circulatory death (DCD) livers, showing that our institutional practice has been to prioritize higher quality organs for the most significantly ill recipients. However, in the current era of machine perfusion, the need to avoid organs traditionally considered marginal for the highest acuity recipients may not be as relevant because of the afforded ability to mitigate ischemia–reperfusion injury and assess organ function before transplantation with normothermic machine perfusion. Going forward, whether it is suitable to increasingly utilize DCD livers for these high-acuity recipients in the era of widespread machine perfusion will certainly be an area of active investigation.^33^

Our multivariate logistic regression model predicted futility in patients with eACLF-3 with an AUROC of 0.74 (SD 0.15), incorporating dynamic changes in oxygenation status along with the novel risk factor of pulmonary pathology as an indication for intubation. We chose to select a decision threshold at a PPV ≥50% in each training set as this corresponds to ∼50% of patients above the threshold experiencing futility. This threshold is associated with a risk score value of 8 and would allow a relatively high specificity and minimization of false positive results with a relatively lower sensitivity (sensitivity: 0.57, specificity 0.81, and PPV 0.45). In the context of selecting patients with eACLF-3 for LT, a relatively lower number of incorrect futility predictions would be desired to not exclude consideration of a patient for receiving a potentially lifesaving LT. Given the relatively low number of events in our cohort, our current model may be overfit to the training data, and a degree of instability is demonstrated by large standard deviations associated with evaluation metrics from cross-validation. Validation in an external dataset is required prior to clinical adoption to predict futility in this population. Although we were able to obtain the MODEL Consortium external validation data, the granularity of data necessary to validate our model was not present. However, key takeaways from these data are that patients with eACLF-3 exist in the LT population with broadly similar characteristics to our cohort, although are rare (n = 169 in 15 centers). Additionally, a similar survival trend was seen where the vast majority of mortality is seen in the short term, with futile outcomes in eACLF-3 observed in 20% of the UCLA cohort and 30% of the MODEL cohort. However, patients with eACLF-3 surviving that first year have acceptable long-term survival, emphasizing that with appropriate patient selection these patients can be transplanted successfully.

### Limitations

Because of the retrospective design of this study, we cannot account for all potential confounding variables, preventing determination of any causal relationships. We focused on patients with eACLF-3 who were transplanted and analyzed risk factors, although we must acknowledge that by design this presents a possible selection bias, whereby the specific subset of listed patients with eACLF-3 surviving to and following transplant differed from patients with eACFL-3 experiencing pre-LT mortality. There were ∼285 patients with eACLF-3 listed for transplant at our center, 108 (38%) of which experienced waitlist dropout without transplant. Additionally, our findings regarding post-transplant survival were broadly corroborated in an external validation cohort. Although the model itself cannot be externally validated because of missing data in the MODEL Consortium data, it should be noted that the main contribution of our manuscript is a description of outcomes in this unique subset of eACLF-3 recipients on maximal pre-LT life support. Our model certainly adds insight into some of the factors that may portend a poor outcome in this subset of patients but will require additional prospective data for validation and testing moving forward. Although our center experience with transplantation of many patients with ACLF-3 and eACLF-3 may not be directly generalizable to other centers with lower acuity patient populations, the similar outcomes observed in the eACFL-3 MODEL population support that acceptable outcomes are achievable with careful patient selection. Futility in LT is not clearly defined and can have differing meanings depending on context.^34^ In this work, we defined futility as 1-year post-transplant death or in-hospital mortality, which is appropriate when considering transplantation for a hyperacute population.

## Conclusions

We report the largest single-center experience of LT for patients with ACLF-3, and define a unique hyperacute cohort of patients with eACLF-3 all of whom required mechanical ventilation, vasopressors, and dialysis before LT. In this subset of patients with eACLF-3, we demonstrate excellent short- and long-term survival considering the high acuity of these patients, which is validated in an external cohort of patients from the MODEL Consortium. We propose novel risk factors associated with futility in this population which may aid in appropriate selection of patients with eACLF-3 requiring maximal life support for lifesaving LT.

## Abbreviations

ACLF, acute-on-chronic liver failure; AIC, Akaike information criterion; ARDS, acute respiratory distress syndrome; CANONIC, Consortium Acute-on-chronic Liver Failure in Cirrhosis; CLIF-C, Chronic Liver Failure Consortium; DCD, donation after circulatory death; eACLF-3, extreme ACLF grade 3; HAT, hepatic artery thrombosis; ICU, intensive care unit; LT, liver transplant; LTACH, long-term acute care hospital; MELD, model for end-stage liver disease; MODEL, Multi-Organ Dysfunction and Evaluation for Liver Transplantation Consortium; MRDO, multi-drug resistant organism; MSOF, multisystem organ failure; OR, odds ratio; OSH, outside hospital; PaO_2_:FiO_2_, partial pressure of oxygen in arterial blood to the fraction of inspiratory oxygen concentration ratio; PE, pulmonary embolism; PNF, primary non-function; PPV, positive predictive value; PRBC, packed red blood cells; RRT, renal replacement therapy; SALT-M, Sundaram ACLF-LT-Mortality; SBP, spontaneous bacterial peritonitis; SNF, skilled nursing facility; TAM, transplantation for ACLF-3 Model; UCLA, University of California, Los Angeles; UNOS, united network for organ sharing; UTI, urinary tract infection.

## Authors’ contributions

Participated in the study concept and design, acquisition of data, interpretation of data, and supervision: all authors. Data collection, analysis, and manuscript writing: AM, AT, DM. The Consortium authors listed at the end of this manuscript were essential collaborators in the acquisition of the multicenter MODEL Consortium data.

## Data availability

The de-identified data are only internally available for the MODEL Consortium researchers after approval of the Ancillary Studies Steering Committee. Single-center data may be available.

## Financial support

RH is a core investigator at the Center for Innovations in Quality, Effectiveness and Safety (CIN 13-413), Michael E. DeBakey VA Medical Center, Houston, TX, USA. KS is supported by the 10.13039/100000002National Institutes of Health (U01CA230690).

## Conflicts of interest

The authors have no conflicts of interest related to this work.

Please refer to the accompanying ICMJE disclosure forms for further details.

## Additional authors from the Multi-Organ Dysfunction and Evaluation for Liver Transplantation (MODEL Consortium)

Atef Al Attar^18^, Kambiz Kosari^18^, Richard Garcia (Departement Hospitalo-Universitaire, Hepatinov, Villejuif, France), Gevork Salmastyan (Departement Hospitalo-Universitaire, Hepatinov, Villejuif, France), William Cranford^6^, Preet Patel^7^, Pei Xue^7^, Soumya Mishra^7^, Madison Parks^7^, Gianina Flocco^8^, Jing Gao^9^, Tiffany Wu^10^, Priya Thanneeru^10^, Vikrant Reddy^11^, Jing Gao^11^, Mariana Hurtado^11^, Islam Mohamed^4^, Ross Vyhmeister^12^, Christine R Lopez^12^, Braidie Campbell^13^, Adam C. Winters^14^, Mary Ann Simpson^15^, Xiaohan Ying (Department of Medicine, Weill Cornell Medicine, New York, NY, USA).
