# Supplementary material for: Liver transplantation at the extreme: Analysis of outcomes and predictors of futility in patients with acute-on-chronic liver failure grade 3 on maximal life support
Source: JHEP Rep. 2026 Aug 5;8(10):101959. doi: 10.1016/j.jhepr.2026.101959 (PMC13613537; doi:10.1016/j.jhepr.2026.101959)
Supplement: Supplementary file 1 [file mmc1.pdf]

Liver transplantation at the extreme: analysis of outcomes and predictors of futility in patients with acute-on-chronic liver failure grade 3 on maximal life support

Andrew Melehy, Asara Thepbunchonchai, Benjamin V. Tran, Ruben Hernaez, Dominic Amara, Samer Ebaid, Daniela Markovic, Fady M. Kaldas, Igor Barjaktarevic, Victor Xia, Constantine J. Karvellas, Saro Khemichian, Lance L. Stein, Kirti Shetty, Christina C. Lindenmeyer, Justin R. Boike, Douglas A. Simonetto, Robert S. Rahimi, Prasun K. Jalal, Manhal Izzy, Michael S. Kriss, Gene Y. Im, Ming V. Lin, Janice H. Jou, Brett E. Fortune, George Cholankeril, Alexander Kuo, Vinay Sundaram, for the Multi-Organ Dysfunction and Evaluation for Liver Transplantation (MODEL) Consortium, Ronald W. Busuttil, Douglas G. Farmer, and Vatche G. Agopian

Table of contents

Table S1……………………………………………………………..……………………..2

Table S2………………………………………………………………………..………..3

Table S3……………………………………………………………..……………………..4

Table S4……………………………………………...………………...……..………...5

Table S5…………………………………………………………….....………....………...6

Fig. S1…………………………………………………………………...…………………..10

BioRender Publication License………………………………………..………….........11

Table S1: Baseline demographic comparisons between extreme and non-extreme ACLF-3 patients

Variable	Total (n=581)	Non-extreme (n=404)	Extreme (n=177)	P
**Demographic**				
Age (y), median (IQR)	57.0 (50.0-63.0)	57.0 (50.0-62.3)	58.0 (49.6-63.9)	0.86
Gender = M (%)	325 (56)	236 (58)	89 (50)	0.06
BMI (kg/m^2), median (IQR)	27.4 (23.7-31.8)	27.7 (23.8-31.9)	26.6 (23.6-31.7)	0.19
Primary Diagnosis (%)				0.24
Alcohol	220 (38)	155 (38)	65 (37)	
MAFLD	115 (20)	70 (17)	45 (25)	
Hepatitis C	89 (15)	55 (14)	34 (19)	
Autoimmune/ cholestasis	54 (9)	33 (8)	21 (12)	
Other	103 (18)	91 (23)	12 (7)	
Previous abdominal surgery	200 (34)	144 (36)	56 (32)	0.49
Diabetes mellitus	163 (28)	109 (27)	54 (31)	0.16
**Pre-LT Acuity**				
MELD-Na at LT, median (IQR)	40.0 (37.0-40.0)	40.0 (38.0-40.0)	40.0 (37.0-40.0)	0.35
Mechanical Ventilation at LT, n (%)	313 (54)	136 (38)	177 (100)	<0.001
Dialysis at LT, n (%)	462 (80)	285 (71)	177 (100)	<0.001
**Pre-LT Hospitalization Characteristics**				
Listing date to LT (d), median (IQR)	17.0 (6.0-127.0)	16.0 (6.0-124.8)	18.0 (6.0-157.0)	0.70
Duration of preoperative admission (d), median	17.0 (10.0-28.0)	16.0 (9.0-25.0)	21.0 (14.0-31.0)	<0.001
ICU admission at LT, n (%)	462 (80)	285 (71)	177 (100)	<0.001
**Donor and Operative Characteristics**				
Donor Risk Index, median (IQR)	1.3 (1.1-1.6)	1.3 (1.1-1.6)	1.3 (1.1-1.6)	0.26
DCD donor	19 (3)	15 (4)	4 (2)	0.77
Cold ischemic time (h), median (IQR)	7.0 (6.0-8.3)	7.2 (6.0-8.4)	7.0 (6.0-8.0)	0.57

ACLF, acute-on-chronic liver failure; IQR, interquartile range; BMI, body mass index; MAFLD, metabolic-associated fatty liver disease; LT, liver transplant; MELD, Model for End-stage Liver Disease; ICU, intensive care unit; DCD, donation after circulatory death.

**Table S2**: Annual center transplant volume and ACLF-3 transplant volume over time with median MELD at transplant in our center and our center's DSA					
Year	Number of liver transplants	Number (%) of ACLF-3 transplants	Number (%) of ACLF-3 transplants for hepatitis C	Median MELD at transplant in southern California donor service area	Median MELD at transplant at our center
2010	164	57 (35)	10 (18)	28	31
2011	180	66 (37)	19 (29)	28	33
2012	144	40 (28)	10 (25)	27	27
2013	159	51 (32)	10 (20)	30	30
2014	155	55 (35)	9 (16)	30	33
2015	139	56 (40)	8 (14)	31	34
2016	151	62 (41)	4 (6)	34	37
2017	135	40 (30)	4 (10)	30	33
2018	153	49 (32)	0 (0)	30	35
2019	157	42 (27)	0 (0)	23	33
2020	158	37 (23)	4 (11)	28	32
2021	136	26 (19)	1 (4)	29	33
ACLF, acute-on-chronic liver failure; MELD, model for end-stage liver disease; DSA, donor service area					

Table S3: Univariable Odds Ratios of Categorical Variables

Variable	OR	Lower 95% CI	Upper 95% CI	P
	.	.	.	.
HCV	6.649	1.330	33.236	0.021
NASH/Cryptogenic/HBV	1.750	0.331	9.250	0.51
EtOH	1.935	0.393	9.534	0.42
Cholestatic/AIH	1.000	.	.	.
Female	1.089	0.520	2.283	0.82
Male	1.000	.	.	.
Previous Lower Abdominal Surgery	1.633	0.602	4.429	0.34
Previous Upper Abdominal Surgery	3.429	1.433	8.203	0.006
No Prior Abdominal Surgery	1.000	.	.	.
Diabetes	0.618	0.261	1.467	0.28
Hyperlipidemia	0.365	0.104	1.277	0.12
Metabolic Syndrome	0.276	0.080	0.956	0.042
Coronary Artery Syndrome	1.262	0.465	3.424	0.65
Sepsis within 1 month	1.018	0.478	2.166	0.96
2+ Sites of infection	2.386	0.708	8.047	0.16
1 Site of infection	2.639	0.832	8.373	0.10
No sights of infection	1.000	.	.	.
Multi-drug resistant infection	1.806	0.591	5.513	0.30
3 or more pressors pre-LT	0.755	0.086	6.638	0.80
2 pressors pre-LT	1.177	0.536	2.585	0.69
No pressors pre-LT	1.000	.	.	.
Intubated for pulmonary pathology	2.931	1.246	6.892	0.014
Intubated for airway protection	1.000	.	.	.
Infection duration > 14 days	1.193	0.276	5.169	0.81
Infection duration 7-14 days	3.303	0.938	11.636	0.06
Infection duration < 7 days	3.418	1.059	11.036	0.04
No infection	1.000	.	.	.

**Table S4:** Univariable Odds Ratios (per SD) for Continuous Measures				
Variable	OR	Lower CL	Upper CL	p-value
Time on waitlist, d	1.257	0.890	1.776	0.19
Age, y	1.023	0.704	1.486	0.90
BMI	0.977	0.673	1.420	0.90
Days admitted prior to LT, d	0.892	0.603	1.320	0.57
Days admitted in ICU prior to LT, d	1.067	0.746	1.527	0.72
PaO_2:FiO_2 ratio at ICU admission	0.921	0.631	1.345	0.67
PEEP at ICU admission	0.596	0.278	1.276	0.18
Log WBC at ICU admission	0.935	0.645	1.354	0.72
Log Plts at ICU admission	1.020	0.704	1.478	0.92
MELD at ICU admission	0.861	0.593	1.250	0.43
MELD Na at ICU admission	0.827	0.567	1.207	0.33
CLIF-C-ACLF at ICU admission	1.094	0.753	1.589	0.64
PaO_2:FiO_2 ratio pre-LT	0.830	0.571	1.205	0.33
PEEP pre-LT	0.989	0.663	1.473	0.96
Base excess pre-LT	0.601	0.414	0.872	0.007
WBC pre-LT	0.984	0.679	1.426	0.93
Plt pre-LT	0.988	0.683	1.432	0.95
MELD pre-LT	1.064	0.731	1.549	0.75
MELD Na pre-LT	1.062	0.729	1.546	0.75
CLIF-C-ACLF pre-LT	1.191	0.813	1.745	0.37
DRI, per unit	1.867	1.069	3.261	0.028
Cold ischemia time	0.920	0.626	1.351	0.67
Change in MELD Na*	1.115	0.753	1.653	0.59
Change in MELD*	1.083	0.744	1.576	0.68
Change in CLIF-C-ACLF*	0.868	0.572	1.317	0.51
Change in WBC*	0.829	0.578	1.188	0.31
Change in Na*	0.806	0.437	1.487	0.49
Change in Plt*	0.848	0.594	1.212	0.37
Change in P:F ratio*	1.071	0.718	1.597	0.74
Percent change in MELD Na	1.147	0.794	1.656	0.47
Increase in MELD Na by at least 6%	2.124	0.944	4.781	0.07
Percent change in MELD	1.099	0.769	1.570	0.61
Percent change in CLIF-C-ACLF	0.858	0.562	1.311	0.48
Percent change WBC	0.937	0.635	1.383	0.74
Percent change Na	0.802	0.432	1.487	0.48
Percent change Plt	0.967	0.646	1.451	0.87
Percent change PaO_2:FiO_2 ratio	1.096	0.769	1.561	0.61
Decrease in PaO_2:FiO_2 ratio by at least 12%	0.464	0.188	1.142	0.10

*Variables denoted by “change” were calculated per SD per day from ICU admission to LT.

**Table S5:** Univariable odds ratios by quartiles of continuous measures				
Variable	OR	lowercl	uppercl	p-value
Time on waitlist, q4	1.5641	0.50221	4.8713	0.44
Time on waitlist, q3	1.5641	0.50221	4.8713	0.44
Time on waitlist, q2	1.15384	0.35457	3.7549	0.81
Time on waitlist, q1	1	.	.	.
Age, q4	0.84416	0.26583	2.6806	0.77
Age, q3	1.47887	0.51488	4.2477	0.47
Age, q2	1.92309	0.68558	5.3944	0.21
Age, q1	1	.	.	.
BMI, q4	1	0.34384	2.9083	1.00
BMI, q3	1.78373	0.6623	4.804	0.25
BMI, q2	0.72499	0.23425	2.2438	0.58
BMI, q1	1	.	.	.
Days admitted prior to LT, q4	0.41316	0.15457	1.1044	0.08
Days admitted prior to LT, q3	0.44946	0.17272	1.1696	0.10
Days admitted prior to LT, q2	0.14607	0.04105	0.5198	0.003
Days admitted prior to LT, q1	1	.	.	.
Days admitted in ICU prior to LT, q4	1.52922	0.51263	4.5618	0.45
Days admitted in ICU prior to LT, q3	0.60673	0.16558	2.2233	0.45
Days admitted in ICU prior to LT, q2	2.77552	0.97317	7.9159	0.06
Days admitted in ICU prior to LT, q1	1	.	.	.
PaO_2:FiO_2 ratio at ICU admission, q4	0.69422	0.20778	2.3195	0.55
PaO_2:FiO_2 ratio at ICU admission, q3	1.67489	0.58737	4.7759	0.33
PaO_2:FiO_2 ratio at ICU admission, q2	1.48359	0.5132	4.2889	0.47
PaO_2:FiO_2 ratio at ICU admission, q1	1	.	.	.
PEEP at ICU admission, q4	0.3012	0.05174	1.7536	0.18
PEEP at ICU admission, q1	1	.	.	.
WBC at ICU admission, q4	0.65714	0.22842	1.8906	0.44
WBC at ICU, admission, q3	1.12794	0.42625	2.9848	0.81
WBC at ICU, admission, q2	0.65714	0.22842	1.8906	0.44
WBC at ICU, admission, q1	1	.	.	.
Platelets at ICU admission, q4	0.87927	0.32149	2.4048	0.80
Platelets at ICU admission, q3	0.85518	0.31319	2.3351	0.76
Platelets at ICU admission, q2	0.65714	0.22842	1.8906	0.44
Platelets at ICU admission, q1	1	.	.	.
CLIF-C-ACLF at LT, q4	1.04638	0.39686	2.7589	0.93
CLIF-C-ACLF at LT, q3	0.62239	0.20389	1.8999	0.41
CLIF-C-ACLF at LT, q2	1.92762	0.71794	5.1755	0.19
CLIF-C-ACLF at LT, q1	1	.	.	.

MELD at ICU admission, q4	0.62441	0.23681	1.6464	0.34
MELD at ICU admission, q3	0.31686	0.10468	0.9591	0.042
MELD at ICU admission, q2	0.54337	0.20134	1.4664	0.23
MELD at ICU admission, q1	1	.	.	.
CLIF-C at ICU admission, q4	1.12794	0.42625	2.9848	0.81
CLIF-C at ICU admission, q3	0.55473	0.18596	1.6548	0.29
CLIF-C at ICU admission, q2	0.76516	0.27356	2.1402	0.61
CLIF-C at ICU admission, q1	1	.	.	.
PaO_2:FiO_2 ratio at LT, q4	0.72632	0.24781	2.1288	0.56
PaO_2:FiO_2 ratio at LT, q3	0.62948	0.20697	1.9145	0.41
PaO_2:FiO_2 ratio at LT, q2	1.50852	0.57229	3.9764	0.41
PaO_2:FiO_2 ratio at LT, q1	1	.	.	.
PEEP at LT, q4	0.88093	0.2014	3.8532	0.87
PEEP at LT, q1	1	.	.	.
Base excess at LT, q4	0.18744	0.06102	0.5758	0.004
Base excess at LT, q3	0.23636	0.07602	0.7349	0.012
Base excess at LT, q2	0.38314	0.14961	0.9812	0.046
Base excess at LT, q1	1	.	.	.
WBC at LT, q4	1.30691	0.46725	3.6555	0.61
WBC at LT, q3	1.43139	0.52003	3.9399	0.49
WBC at LT, q2	0.72499	0.23425	2.2438	0.58
WBC at LT, q1	1	.	.	.
Platelets at LT, q4	1.14996	0.40271	3.2837	0.79
Platelets at LT, q3	1.00447	0.35441	2.8469	0.99
Platelets at LT, q2	1.14996	0.40271	3.2837	0.79
Platelets at LT, q1	1	.	.	.
MELD at LT, q4	1	0.34384	2.9083	1.00
MELD at LT, q3	0.97333	0.33516	2.8266	0.96
MELD at LT, q2	1.47412	0.5346	4.0647	0.45
MELD at LT, q1	1	.	.	.
CLIF-C-ACLF, q4	1.80267	0.6043	5.3775	0.29
CLIF-C-ACLF, q3	1.54161	0.50912	4.668	0.44
CLIF-C-ACLF, q2	1.80267	0.6043	5.3775	0.29
CLIF-C-ACLF, q1	1	.	.	.
Cold ischemia time, q4	0.84433	0.29517	2.4152	0.75
Cold ischemia time, q3	1.13928	0.41353	3.1387	0.80
Cold ischemia time, q2	0.62798	0.20583	1.9159	0.41
Cold ischemia time, q1	1	.	.	.
MELD change ICU to LT, q4	1.92309	0.68558	5.3944	0.21
MELD change ICU to LT, q3	1.13333	0.37978	3.382	0.82
MELD change ICU to LT, q2	1.16438	0.38963	3.4797	0.79

MELD change ICU to LT, q1	1	.	.	.
CLIF-C ACLF change ICU to LT, q4	1.28281	0.47593	3.4577	0.62
CLIF-C ACLF change ICU to LT, q3	0.5203	0.16364	1.6544	0.27
CLIF-C ACLF change ICU to LT, q2	1.13731	0.41583	3.1105	0.80
CLIF-C ACLF change ICU to LT, q1	1	.	.	.
WBC change ICU to LT, q4	1.33802	0.45725	3.9154	0.60
WBC change ICU to LT, q3	1.92309	0.68558	5.3943	0.21
WBC change ICU to LT, q2	1	0.32587	3.0687	1.00
WBC change ICU to LT, q1	1	.	.	.
Na change ICU to LT, q4	0.65714	0.22842	1.8906	0.44
Na change ICU to LT, q3	0.52381	0.16614	1.6515	0.27
Na change ICU to LT, q2	1.18286	0.46225	3.0268	0.73
Na change ICU to LT, q1	1	.	.	.
Platelets change ICU to LT, q4	1.26375	0.48346	3.3034	0.63
Platelets change ICU to LT, q3	0.85518	0.31319	2.3351	0.76
Platelets change ICU to LT, q2	0.36502	0.10911	1.2211	0.10
Platelets change ICU to LT, q1	1	.	.	.
PaO_2:FiO_2 ratio change ICU to LT, q4	0.76259	0.27075	2.1479	0.61
PaO_2:FiO_2 ratio change ICU to LT, q3	0.76259	0.27075	2.1479	0.61
PaO_2:FiO_2 ratio change ICU to LT, q2	0.65392	0.22579	1.8939	0.43
PaO_2:FiO_2 ratio change ICU to LT, q1	1	.	.	.
Percent change MELD ICU to LT, q4	1.92309	0.68558	5.3944	0.21
Percent change MELD ICU to LT, q3	0.97403	0.31783	2.985	0.96
Percent change MELD ICU to LT, q2	1.33802	0.45725	3.9154	0.60
Percent change MELD ICU to LT, q1	1	.	.	.
Percent change CLIF-C-ACLF ICU to LT, q4	1.28281	0.47593	3.4577	0.62
Percent change CLIF-C-ACLF ICU to LT, q3	0.6309	0.20805	1.9132	0.42
Percent change CLIF-C-ACLF ICU to LT, q2	1	0.35917	2.7842	1.00
Percent change CLIF-C-ACLF ICU to LT, q1	1	.	.	.
Percent change WBC ICU to LT, q4	1.52174	0.52894	4.378	0.44
Percent change WBC ICU to LT, q3	1.92309	0.68558	5.3943	0.21
Percent change WBC ICU to LT, q2	0.84416	0.26583	2.6806	0.77
Percent change WBC ICU to LT, q1	1	.	.	.
Percent change bilirubin ICU to LT, q4	2.35294	0.73260	7.55705	0.15
Percent change bilirubin ICU to LT, q3	2.05714	0.62984	6.71886	0.23
Percent change bilirubin ICU to LT, q2	2.66666	0.84163	8.44916	0.10
Percent change bilirubin ICU to LT, q1	1	.	.	.
Percent change INR ICU to LT, q4	0.90909	0.34138	2.42092	0.85
Percent change INR ICU to LT, q3	0.79481	0.29257	2.15919	0.65
Percent change INR ICU to LT, q2	0.39627	0.12512	1.25503	0.12
Percent change INR ICU to LT, q1	1	.	.	.

Percent change Na, ICU to LT, q4	0.65714	0.22842	1.8906	0.44
Percent change Na, ICU to LT, q3	0.52381	0.16614	1.6515	0.27
Percent change Na, ICU to LT, q2	1.18286	0.46225	3.0268	0.73
Percent change Na, ICU to LT, q1	1	.	.	.
Percent change platelets, ICU to LT, q4	0.87927	0.32149	2.4048	0.80
Percent change platelets, ICU to LT, q3	0.85518	0.31319	2.3351	0.76
Percent change platelets, ICU to LT, q2	0.65714	0.22842	1.8906	0.44
Percent change platelets, ICU to LT, q1	1	.	.	.
Percent change PaO_2:FiO_2 ratio ICU to LT, q4	0.77676	0.286	2.1096	0.62
Percent change PaO_2:FiO_2 ratio ICU to LT, q3	0.48779	0.16464	1.4452	0.20
Percent change PaO_2:FiO_2 ratio ICU to LT, q2	0.57868	0.20255	1.6533	0.31
Percent change PaO_2:FiO_2 ratio ICU to LT, q1	1	.	.	.

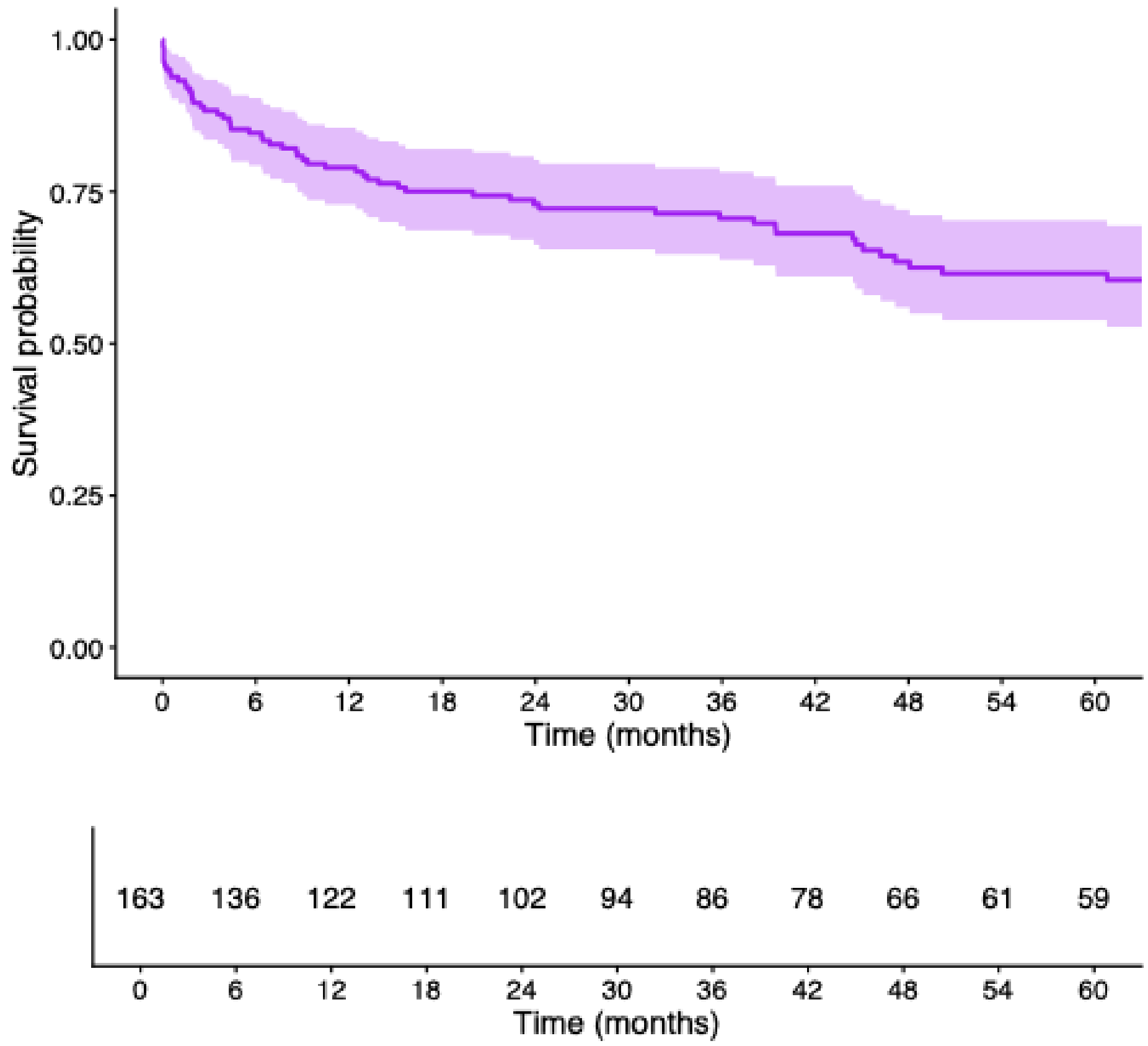

Fig. S1: Overall post-transplant survival in extreme ACLF-3 patients excluding those receiving a simultaneous liver-kidney transplant (n=14).

49 Spadina Ave. Suite 200
Toronto ON M5V 2J1 Canada
www.biorender.com

Confirmation of Publication and Licensing Rights - Open Access

December 11th, 2025

Subscription Type:	*Postdoc Plan - Academic*
Agreement number:	*QW293RUI0C*
Publisher Name:	*JHEP Reports*
Figure Title:	*Graphical Abstract*
Citation to Use:	*Created in BioRender. Melehy, A. (2026) https://BioRender.com/t59ytz1*

To whom this may concern,

This document ("Confirmation") hereby confirms that Science Suite Inc. dba BioRender ("BioRender") has granted the following BioRender user: Andrew Melehy ("User") a BioRender Academic Publication License in accordance with BioRender's Terms of Service and Academic License Terms ("License Terms") to permit such User to do the following on the condition that all requirements in this Confirmation are met:

1) publish their Completed Graphics created in the BioRender Services containing both User Content and BioRender Content (as both are defined in the License Terms) in publications (journals, textbooks, websites, etc.); and
2) sublicense such Completed Graphics under "open access" publication sublicensing models such as CC-BY 4.0 and more restrictive models, so long as the conditions set forth herein are fully met.

Requirements of User:

1) All Completed Graphics to be published in any publication (journals, textbooks, websites, etc.) must be accompanied by the following citation either as a caption, footnote or reference for each figure that includes a Completed Graphic:
"Created in BioRender. Melehy, A. (2026) https://BioRender.com/t59ytz1 ".
2) All terms of the License Terms including all Prohibited Uses are fully complied with. E.g. For Academic License Users, no commercial uses (beyond publication in journals, textbooks or websites) are permitted without obtaining or switching to a BioRender Industry Plan.
3) A Reader (defined below) may request that the User allow their figure to be a public template for Readers to view, copy, and modify the figure. It is up to the User to determine what level of access to grant.

Open-Access Journal Readers:

Open-Access journal readers ("Reader") who wish to view and/or re-use a particular Completed Graphic in an Open-Access journal subject to CC-BY sublicensing may do so by clicking on the URL link in the applicable citation for the subject Completed Graphic.

The re-use/modification options below are available after the Reader requests the User to adapt their
