# Supplementary material for: Liver transplantation at the extreme: Analysis of outcomes and predictors of futility in patients with acute-on-chronic liver failure grade 3 on maximal life support
Source: JHEP Rep. 2026 Aug 5;8(10):101959. doi: 10.1016/j.jhepr.2026.101959 (PMC13613537; doi:10.1016/j.jhepr.2026.101959)
Supplement: Supplementary file 3 [file mmc4.pdf]

Research article

JHEP|Reports

# Liver transplantation at the extreme: Analysis of outcomes and predictors of futility in patients with acute-on-chronic liver failure grade 3 on maximal life support

## Authors

Andrew Melehy, Asara Thepbunchonchai, Benjamin V. Tran, …, Ronald W. Busuttil, Douglas G. Farmer, Vatche G. Agopian

## Correspondence

vagopian@mednet.ucla.edu (V.G. Agopian).

## Graphical abstract

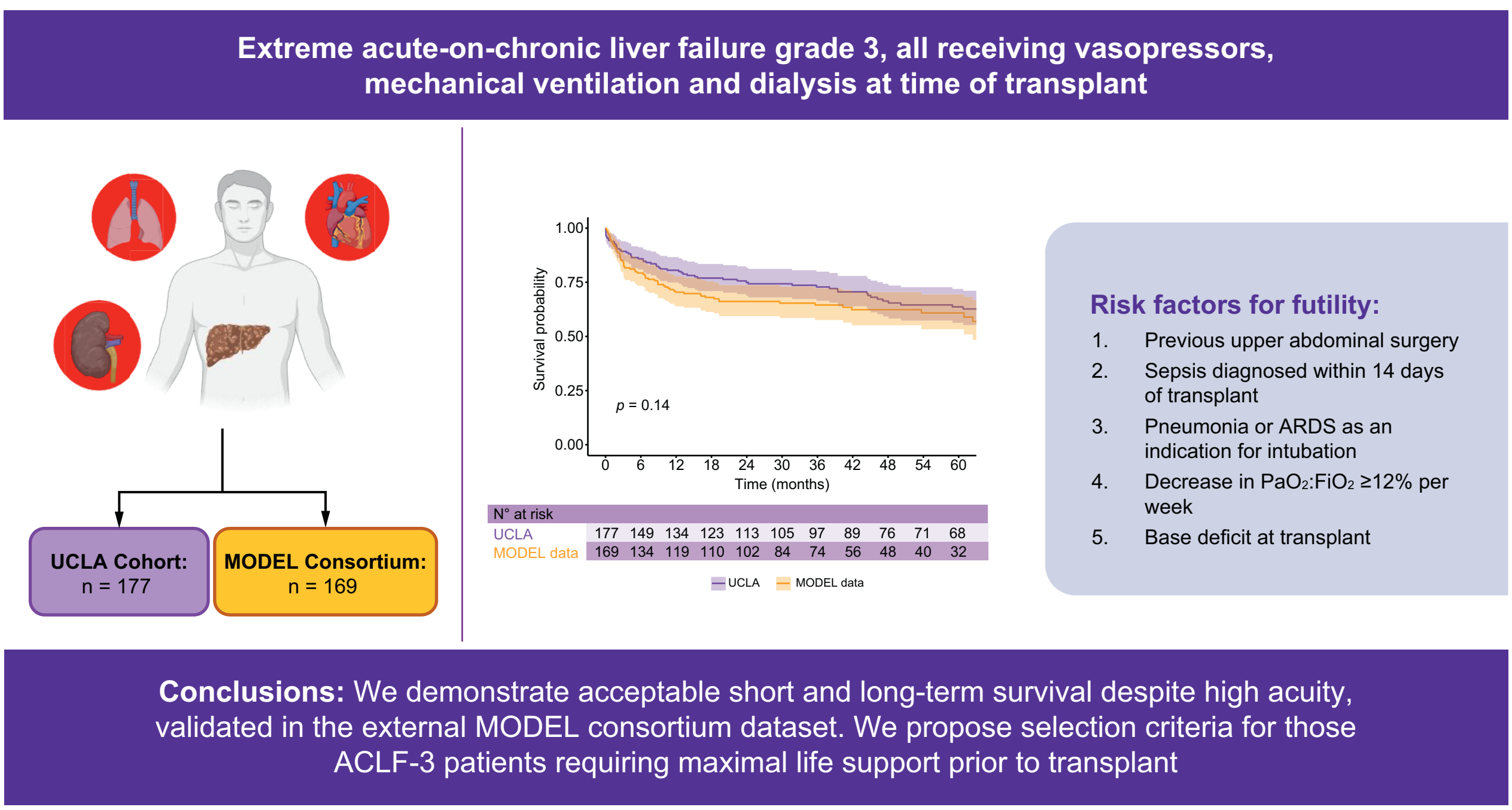


## Highlights:

- LT recipients with ACLF-3 on maximal life support represent a unique, high-acuity transplant population.
- One-year mortality is 20%, but long-term survival is acceptable among patients surviving the first year.
- Sepsis within 2 weeks before transplant is a risk factor for futility in this high-acuity subpopulation.
- Intubation because of pneumonia or acute respiratory distress syndrome and worsening $PaO_2$:$FiO_2$ ratio predict futility.

## Impact and implications:

There has been persistent interest in the liver transplant community regarding transplant for acute-on-chronic liver failure grade 3 (ACLF-3) recipients. In this work, we report on a large single-center experience transplanting ACLF-3 recipients, specifically investigating an extreme subset of these patients who were all mechanically ventilated, receiving vasopressors, and receiving hemodialysis at time of transplant. We characterized their outcomes, identifying clear risk factors associated with futility in this hyperacute subgroup and demonstrating that with appropriate selection, even patients in this extreme subgroup can experience acceptable 5-year post-transplant survival. Although we could not externally validate our model because of data availability, the obtained external patient cohort of patients with extreme ACLF-3 broadly validates our findings. We feel that these results represent an important contribution to the liver transplant community given concerns about transplanting this hyperacute subpopulation. These results may help guide physicians in selecting appropriate candidates for transplant.

https://doi.org/10.1016/j.jhepr.2026.101959
© 2026 The Author(s). Published by Elsevier B.V. on behalf of European Association for the Study of the Liver (EASL). This is an open access article under the CC BY license (http://creativecommons.org/licenses/by/4.0/). 

JHEP|Reports

# Liver transplantation at the extreme: Analysis of outcomes and predictors of futility in patients with acute-on-chronic liver failure grade 3 on maximal life support

Andrew Melehy[1], Asara Thepbunchonchai[1], Benjamin V. Tran[1], Ruben Hernaez[2,3,4], Dominic Amara[1], Samer Ebaid[1], Daniela Markovic[1], Fady M. Kaldas[1], Igor Barjaktarevic[1], Victor Xia[1], Constantine J. Karvellas[5], Saro Khemichian[6], Lance L. Stein[7], Kirti Shetty[8], Christina C. Lindenmeyer[9], Justin R. Boike[10], Douglas A. Simonetto[11], Robert S. Rahimi[12], Prasun K. Jalal[5], Manhal Izzy[13], Michael S. Kriss[14], Gene Y. Im[15], Ming V. Lin[16], Janice H. Jou[17], Brett E. Fortune[18], George Cholankeril[5], Alexander Kuo[19], Vinay Sundaram[19,†], for the Multi-Organ Dysfunction and Evaluation for Liver Transplantation (MODEL) Consortium, Ronald W. Busuttil[1], Douglas G. Farmer[1], Vatche G. Agopian[1,*]

JHEP Reports **2026**. vol. 8 | 1–11

Check for updates

**Background & Aims:** Although patients with grade 3 acute-on-chronic liver failure (ACLF-3), have high mortality, there is a subset requiring maximal life support. We report post liver transplant (LT) outcomes and identify factors associated with futility in extreme ACLF-3 (eACLF-3) recipients, all requiring intubation, vasopressors, and dialysis at LT.

**Methods:** We identified adult eACLF-3 LT recipients between January 2010 and December 2021. Pre-LT characteristics were compared among eACLF-3 recipients with and without the futile outcome (1-year or in-hospital mortality), predictors of futility were identified, and a logistic regression model was evaluated using fivefold cross-validation. Findings were validated using the Multi-Organ Dysfunction and Evaluation for Liver Transplantation (MODEL) Consortium.

**Results:** Of 1,608 adult LT recipients at our center, 177 (11%) were eACLF-3. Thirty-five (20%) eACLF-3 recipients experienced futility. Predictors of futility were previous upper abdominal surgery (odds ratio [OR] = 4.6, $p$ = 0.002), sepsis within 14 days before LT (OR = 5.9, $p$ = 0.013), intubation because of pneumonia or acute respiratory distress syndrome (OR = 3.5, $p$ = 0.010), worsening $PaO_2$:$FiO_2$ ratio (OR = 3.9, $p$ = 0.008), and pre-LT base deficit (OR = 1.27, $p$ = 0.007). Futility was predicted with an AUROC of 0.74 (SD 0.15). When compared with the MODEL Consortium, our cohort had improved 3-, 6-, and 12-month survivals of 89%, 85%, and 80%, compared with 83%, 79%, and 70%, respectively ($p$ = 0.048); however overall 5-year survival was similar (63% *vs.* 59%, $p$ = 0.14).

**Conclusions:** We report the largest single-center experience of LT for patients with eACLF-3, demonstrating acceptable survival despite high acuity, validated in the MODEL Consortium dataset. Prospective validation of the predictive model and identified risk factors is necessary, and may aid in appropriate selection of patients with ACLF-3 requiring maximal life support before LT.

© 2026 The Author(s). Published by Elsevier B.V. on behalf of European Association for the Study of the Liver (EASL). This is an open access article under the CC BY license (http://creativecommons.org/licenses/by/4.0/).

## Introduction

Acute-on-chronic liver failure (ACLF) is a syndrome characterized by acute hepatic decompensation, organ failure, and high short-term mortality.[1–5] Patients with cirrhosis who develop an acute decompensation are at risk for the development of ACLF with subsequent rapid organ dysfunction and an elevated mortality risk.[6–8] The European CANONIC trial (Consortium Acute-on-chronic Liver Failure in Cirrhosis) defined ACLF in three grades according to the number of associated organ failures.[1,9] ACLF can be stratified using the Chronic Liver Failure Consortium (CLIF)-Organ failure score, which reflects the number and degree of organ failures and the CLIF-C ACLF score which predicts outcomes in patients with ACLF and may better assess risk of waitlist mortality in patients with ACLF than the model for end-stage liver disease (MELD) score.[10] ACLF grade 3 (ACLF-3) is the most severe form of the disease where patients have at least three organ failures and is associated with 28-day waitlist mortality approaching 80%.[7,10–13] In an analysis of the United Network for Organ Sharing (UNOS) database, ACLF-3 recipients had worse waitlist and post-transplant survival compared with status 1A recipients, traditionally considered to be the highest acuity liver transplant (LT) population.[12]

Because of the extremely high waitlist mortality of patients with ACLF-3, there is considerable interest in offering these patients early LT to afford them the greatest chance of survival.[14–21] However, this is balanced against the concerns about poor post-transplant outcomes and stewardship of

* Corresponding author. Address: Division of Liver and Pancreas Transplantation, Department of Surgery, David Geffen School of Medicine at UCLA, Los Angeles, CA, USA.
*E-mail address:* vagopian@mednet.ucla.edu (V.G. Agopian).
† Deceased.
https://doi.org/10.1016/j.jhepr.2026.101959

scarce donor livers. There have thus been considerable efforts to predict futility following transplant in ACLF-3 recipients and to identify risk factors for futile outcomes.[7,22–25] In one large multicenter ACLF-3 study, five European centers contributed data on 152 ACLF-3 LT recipients and created the 'transplantation for ACLF-3 model' (TAM score) to predict 1-year post-transplant mortality.[24] This study identified risk factors for 1-year post-transplant mortality including advanced age, lactate, respiratory failure, and increasing white blood cell count and were able to predict 1-year mortality with an AUROC of 0.88. Another recent study was reported by Hernaez *et al.*[25] where 15 US centers contributed data on 735 patients with ACLF, 521 of whom were considered 'severe', including patients with ACLF 2 and 3. These data were used to create the novel Sundaram ACLF-LT-Mortality (SALT-M) score, which predicts 1-year post-transplant mortality in this group of patients with severe ACLF with a reported AUROC of 0.80.

Although the TAM and SALT-M scores represent significant contributions to the ability to discriminate patients with severe ACLF who may be at risk for 1-year post-transplant mortality, there is a spectrum of disease even within ACLF-3 that has not been specifically explored. The European definition of ACLF, based on the CLIF-C organ failure scoring system, assesses function of six organ systems (liver, kidney, brain, coagulation, circulation, and respiration).[10] Based on this system, patients can be considered to have ACLF-3 with evidence of failure in any three of these organ systems. It is likely that a patient with acutely decompensated liver function, disordered coagulation, and hepatic encephalopathy would have a significantly different risk profile compared to a patient with respiratory failure requiring mechanical ventilation, circulatory failure requiring vasopressors, and renal failure requiring dialysis.[26] In the original TAM score study, 73% of the cohort was mechanically ventilated, 70% on pressors, and 65% receiving dialysis at time of transplant. In the cohort used to create the SALT-M score, 48% were receiving mechanical ventilation, 52% were on vasopressors, and 84% were receiving dialysis at time of LT. We hypothesized that patients with ACLF-3 requiring concomitant mechanical ventilation, vasopressors, and dialysis – all forms of critical life support – represent a hyperacute subgroup of extreme ACLF-3 (eACLF-3) transplant recipients who have not specifically been analyzed in prior studies. We sought to characterize our single-center experience in transplanting these patients, with a specific goal of identifying risk factors for futility after transplant. Additionally, we sought to externally validate our findings using data from the Multi-Organ Dysfunction and Evaluation for Liver Transplantation (MODEL) Consortium, which was used to develop the SALT-M score.[25]

## Patients and methods

### Patient cohorts

We identified adult (age ≥18 years), first-time, non-status 1A, liver-only or liver–kidney transplant recipients at UCLA Medical Center from January 1, 2010 to December 31, 2021 through retrospective chart review. After exclusion, we identified patients with decompensation with one or more additional organ failures and stratified them based on ACLF score (1, 2, or 3). Within the patients with ACLF-3, we specifically identified recipients with acute decompensation of cirrhosis with multiple organ failures resulting in the need for mechanical ventilation, vasopressors, and renal replacement therapy (eACLF-3) at the time of transplantation. This study was approved by the UCLA institutional review board (IRB #11-001932).

Data collection for the MODEL Consortium has been previously described,[25] approval from contributing authors was obtained and data had been collected under IRB approvals at each participating institution. From this cohort, we identified eACLF-3 LT recipients requiring vasopressors, on dialysis, and mechanically ventilated at time of LT.

### Data collection and definition of eACLF-3

Patients meeting the criteria for ACLF (grades 1–3) were identified and categorized at the time of LT based on the EASL-CLIF criteria. The patients with severe ACLF-3 were then further stratified into eACLF-3, which included only those who were mechanically ventilated, receiving vasopressors, and receiving renal replacement therapy at the time of LT. If patients were extubated, weaned off pressors, or weaned off dialysis before LT, they were not included as part of this eACLF-3 cohort. For the eACLF-3 cohort, pre-transplant clinical characteristics were collected at time of intensive care unit (ICU) admission and at time of transplant. Several variables were calculated using the change in value from ICU admission to transplant, including change in MELD score and change in the ratio of the partial pressure of oxygen in arterial blood to the fraction of inspiratory oxygen concentration ($PaO_2$:$FiO_2$ ratio). The indication for mechanical ventilation was recorded as either strictly for airway protection or due to pulmonary pathology including pneumonia and acute respiratory distress syndrome (ARDS). The indication for ventilation was determined through manual chart review with physician review of chest X-rays, X-ray radiology reports, and any relevant microbiological data including sputum and blood cultures. Sepsis was considered if patients had clinical suspicion for sepsis with an identified (culture proven) infectious source (pneumonia, spontaneous bacterial peritonitis, urinary tract infection, or other source). We included a sepsis duration variable that signified if a patient was diagnosed with sepsis longer than 2 weeks before transplant, within 2 weeks, or not diagnosed with sepsis.

### Outcomes

The primary outcome of interest was futility following transplant, defined as 1-year post-transplant all-cause mortality or in-hospital mortality. We included in-hospital mortality in the definition to capture patients who receive prolonged intensive care and life support following transplant, ultimately experiencing mortality in the hospital, which should be considered a futile outcome even if it did not occur within 1 year. Extensive secondary outcomes were evaluated and reported, including 5-year overall survival, post-LT ICU and total length of hospitalization, total time of intubation, number of reoperations, planned *vs*. unplanned reoperations, time to reoperation, postoperative respiratory failure (requiring re-intubation), neurological failure (ischemic stroke or cerebral hemorrhage), cardiac failure (heart failure leading to low output syndrome), sepsis, portal vein thrombosis, hepatic artery thrombosis, primary graft non-function, discharge to skilled nursing facility, discharge to long-term acute care hospital, readmission within

1 month after discharge, dialysis dependence following transplant, tracheostomy-dependence, graft failure requiring re-transplant, and cause of death.

### Statistical analysis

Analyses were conducted using SAS (SAS Institute, Cary, NC, USA) and R version 4.1.0 (R Foundation for Statistical Computing, Vienna, Austria). Any *p* values <0.05 were considered significant.

Baseline characteristics at time of ICU admission and at LT were compared between the futile and non-futile groups. Characteristics were also compared between patients who were eACLF-3 and non-eACLF-3 at our center as well as patients with eACLF-3 at our center and in the MODEL Consortium. Group medians were displayed with IQR ranges and compared using the Mann–Whitney *U* test. Categorical variables were displayed as number (%) and compared using the $X^2$ test. We compared survivals between the patients with futile and non-futile eACLF-3 as well as the patients with eACLF-3 at our center and in the MODEL Consortium using the Kaplan–Meier method. Although our focus was on patients with eACLF-3, we also compared overall survival between patients with ACLF 1, 2, and 3 using the Kaplan–Meier method. Survival differences were assessed for statistical significance using the log-rank test in all comparisons.

Univariate logistic regression analysis with futility modeled as the outcome was conducted for all collected variables. For features that were calculated based on change in values from ICU admission to LT, we rigorously tested different binarized threshold value cut-offs and determined quantities of change most predictive of futility empirically. Features for the final multivariate logistic regression model were selected using stepwise selection based on the Akaike information criterion (AIC) and clinical relevance. Model performance was assessed using fivefold cross-validation on the internal UCLA data with each training-validation split stratified on the futility outcome. A decision threshold was selected at the lowest predicted probability resulting in a positive predictive value (PPV) of ≥50% in each training fold. Using each training probability threshold, sensitivity, specificity, and PPV were calculated on each validation fold. AUROC was also calculated across the five folds and all evaluation metrics were reported with mean and standard deviation. Model calibration was assessed using the Brier score and the Hosmer–Lemeshow test. A risk score was created by dividing each feature coefficient by the smallest coefficient value and rounding to the nearest integer. The median predicted probability used as a decision threshold in each training fold was used to determine the risk score threshold over which futility should be strongly considered. Missing values for variables in the final multivariate model were imputed using multiple imputation by chained equations using the ‘MICE’ R package (R Foundation for Statistical Computing).[27]

#### *Removal of simultaneous liver–kidney transplant recipients*

Patients receiving simultaneous liver–kidney transplant were removed in this sensitivity analysis. Post-transplant survival was plotted using the Kaplan–Meier method and a multivariate logistic regression model was created and evaluated using the same procedures as above.

## Results

Of 2,024 total LTs performed at our center, 1,608 met study inclusion criteria, of which 182 (11%) were ACLF-1, 250 (16%) were ACLF-2, and 581 (36%) were for ACLF-3 (Fig. 1). The remaining 595 (37%) patients were those with hepatocellular carcinoma (369, 23%), non-HCC related MELD exception points (117, 7%), or decompensated cirrhosis not requiring hospitalization before transplant (109, 7%). Of the 581 patients with ACLF-3, 177 (30%) represented patients with eACLF-3 all requiring mechanical ventilation, vasopressors, and receiving renal replacement therapy at time of LT. Baseline demographic comparisons between patients with non-extreme ACLF-3 and eACLF-3 are shown in Table S1. Annual transplant center volume, number (%) of transplants for ACLF-3, and transplant center and donor service area median MELD at transplant during successive years of the study period are shown in Table S2. There were 35 patients with eACLF-3 who experienced the futile outcome (20%), with baseline characteristics shown in Table 1. Compared with patients with non-futile eACLF-3, those with futile eACLF-3 were significantly more likely to have hepatitis C (43% *vs*. 21%, *p* = 0.012), previous upper abdominal surgery (13% *vs.* 11%, *p* = 0.042), had a greater base deficit at time of transplant (median, IQR: 0.0, −2.0–1.0 *vs*. 1.0, −1.0–2.0, *p* = 0.004), were more likely to be intubated at LT for pneumonia or ARDS (77% *vs.* 54%, *p* = 0.011), and were more likely to have sepsis within 14 days before transplant (77% *vs.* 54%, *p* = 0.040). The two groups did not significantly differ in MELD or CLIF-C ACLF scores at ICU admission or at transplant, and had similar waitlist times, duration of pre-transplant total and ICU admission, and similar proportion of outside hospital admission and duration of admission before transferring care to our center. Regarding donor and operative characteristics, patients with futile eACLF-3 received liver allografts with greater median donor risk index (median, IQR: 1.4, 1.2–2.3 *vs.* 1.3, 1.1–1.6, *p* = 0.011), received more packed red blood cell transfusions during transplant (median, IQR: 31.0 (18.0–50.0) *vs.* 23.0 (15.0–36.0), *p* = 0.049), and were more likely to have required venovenous bypass during transplant (86% *vs.* 65%, *p* = 0.017) compared with the non-futile eACLF-3 cohort.

Overall survival was decreased in patients with ACLF-3 compared with those with ACLF-1 and ACLF-2 (though not statistically significantly), with survivals of 87%, 90%, and 83% at 1-year, 74%, 81%, and 76% at 3-years, and 70%, 74%, and 69% at 5 years for patients with ACLF-1, ACLF-2, and ACLF-3, respectively (Fig. 2A, overall *p* = 0.19). In a sub-analysis of patients with ACLF-3, patients with eACFL-3 had lower 1-, 3-, and 5-year survival compared with non-eACLF-3 (81%, 73%, and 63% *vs.* 84%, 77%, and 71%, respectively; Fig. 2B, *p* = 0.013).

Details of the causes of death and comparisons of secondary outcomes between the eACLF-3 non-futile and futile groups are shown in Table 2. Patients in the futile group had a longer median time of post-LT intubation (45.0 days, IQR: 7.0–94.0 *vs.* 8.0 days, IQR: 3.0–27.5, *p* = 0.003), and a larger proportion of patients with two or greater unplanned reoperations (20% *vs*. 11%, *p* = 0.025). Additionally, patients in the futile group were more likely to have had postoperative respiratory, neurological, and cardiac failure (97%, 60%, 69% *vs.* 59%, 9%, 10%, *p* <0.001 for all comparisons). In the futile group, the most common cause of death within 1 year was sepsis with multisystem organ failure (23%).

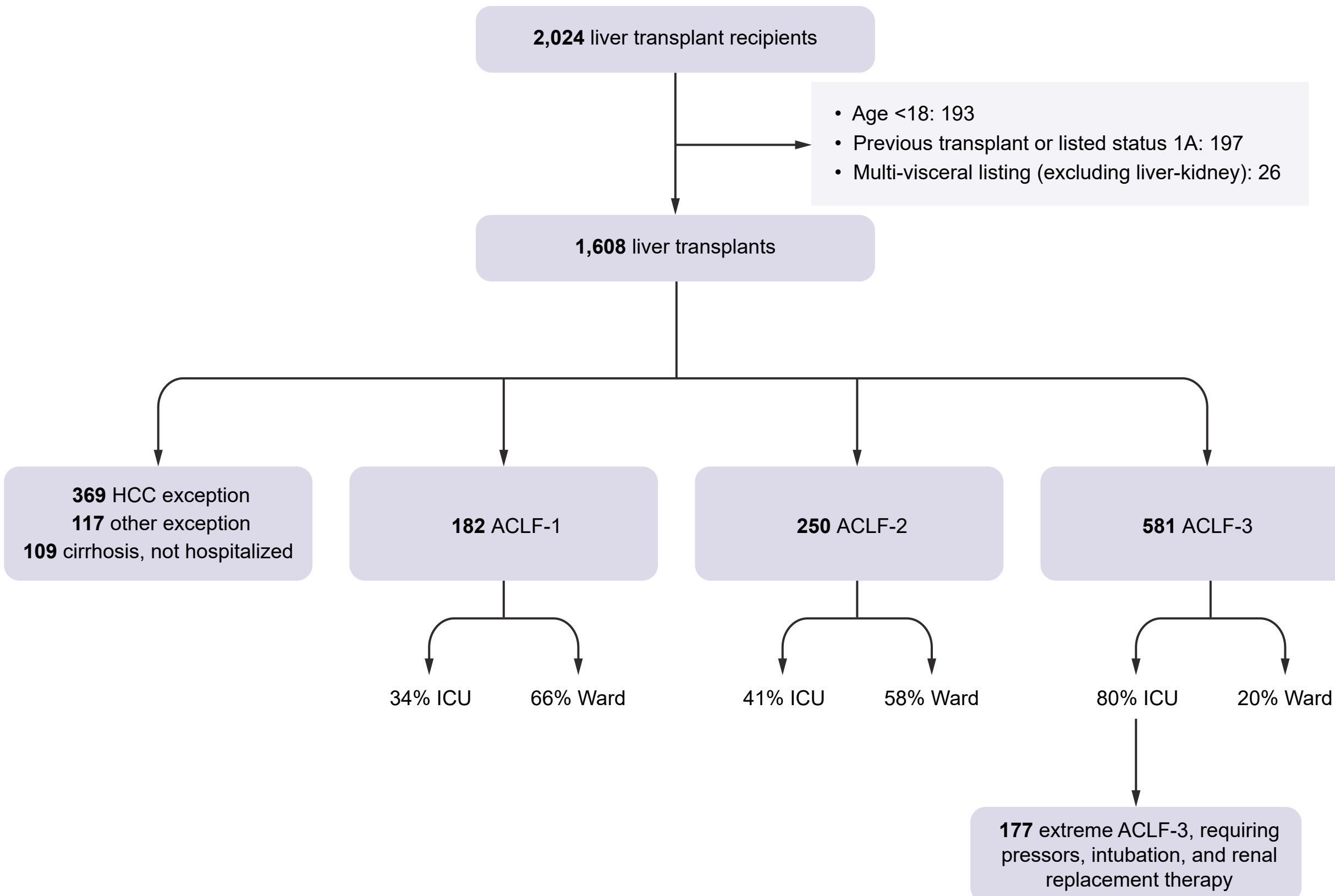


**Fig. 1. Selection of patient cohorts, with exclusions made for pediatric patients, those with previous transplants, status 1A recipients, multi-visceral recipients (excluding liver–kidney), and transplants outside the study timeframe.** The extreme ACLF-3 cohort was identified from among the patients with ACLF-3 who were transplanted.

Univariate modeling results evaluating all candidate factors associated with the futile outcome are detailed in Table S3 (categorical variables) and Table S4 (continuous variables). Tested thresholds for continuous variables converted to categorical factors are shown in Table S5. The final selected multivariate logistic regression model from stepwise selection had an AIC of 158 with complete model results is shown in Table 3. The final selected model features for post-LT futility included previous upper abdominal surgery (odds ratio [OR] = 4.6; 95% CI, 1.8–12.1, $p$ = 0.002), sepsis within 14 days before LT (OR = 5.9; 95% CI, 1.5–24.1, $p$ = 0.013), requiring ventilatory support as a result of pneumonia or ARDS (OR = 3.5; 95% CI, 1.4–9.2, $p$ = 0.010), worsening $PaO_2$:$FiO_2$ ≥12% per week (OR = 3.9; 95% CI, 1.4–10.6, $p$ = 0.008), and increasing pre-LT base deficit (OR = 1.27; 95% CI, 1.1–1.5; $p$ = 0.007). The Brier score for the final model was 0.12 and Hosmer–Lemeshow $p$ value was 0.38 (≥0.05 suggests no significant differences between observed and predicted values). On fivefold cross-validation, the final model predicted futility in patients with eACLF-3 with a mean sensitivity of 0.57 (SD 0.23), specificity of 0.81 (SD 0.11), PPV of 0.45 (SD 0.17), and AUROC of 0.74 (SD 0.15). The median decision threshold used was a predicted probability of 0.25. The reported risk score can be calculated using values in Table 3 and the identified risk score threshold was 8, corresponding to a predicted probability of 0.25, above which futility occurred in ~50% of patients.

### Removal of simultaneous liver–kidney transplant recipients

Overall post-transplant survival was similar when simultaneous liver–kidney transplant recipients were removed from analysis with 1-, 3-, and 5-year survival of 78%, 71%, and 61%, respectively; Fig. S1). Overall, the logistic regression modeling efforts showed no significant differences when simultaneous liver–kidney transplant recipients were excluded. The same five features were selected for the final model in stepwise regression. The median selected decision threshold was at a predicted probability of 0.26. The average AUROC was 0.77 (SD 0.13), sensitivity 0.66 (SD 0.13), specificity 0.77 (0.12), and PPV 0.48 (SD 0.22).

### Comparison to external MODEL Consortium cohort

There were 169 patients meeting criteria for eACLF-3 in the MODEL Consortium dataset. Complete comparisons of demographics and outcomes are shown in Table 4. In terms of outcomes, MODEL patients had shorter median post-LT ICU lengths of stay (9 days *vs.* 29 days, $p$ <0.001), shorter median total lengths of stay (22 days *vs.* 53 days, $p$ <0.001), shorter median duration of post-LT intubation (2 days *vs.* 9 days, $p$ <0.001), fewer re-transplants (1% *vs.* 6%, $p$ = 0.017) compared to the UCLA cohort. However, MODEL eACFL-3 patients experienced more readmissions 1 month after LT (36% *vs.*

**Table 1. Baseline demographic comparisons between patients with ACLF grade 3 with and without the futile outcome.**

| Variable | Total (N = 177) | Non-futile (n = 142) | Futile (n = 35) | *p* values |
|---|---|---|---|---|
| **Demographic** | | | | |
| Age (y) | 58.0 (49.6–64.0) | 57.6 (51.9–63.3) | 58.3 (49.1–64.1) | 0.70 |
| Sex = M | 89 (50) | 72 (51) | 17 (49) | 0.82 |
| BMI (kg/m$^2$) | 26.6 (23.6–31.7) | 26.5 (23.4–31.8) | 27.8 (24.1–31.4) | 0.79 |
| Primary diagnosis | | | | 0.012 |
| Alcohol | 65 (37) | 54 (38) | 11 (31) | |
| MASLD | 45 (25) | 38 (27) | 7 (20) | |
| Hepatitis C | 34 (19) | 20 (14) | 14 (40) | |
| Autoimmune/cholestasis | 21 (12) | 19 (14) | 2 (6) | |
| Other | 12 (7) | 10 (7) | 2 (6) | |
| Prior abdominal surgery | 70 (40) | 50 (35) | 20 (57) | 0.018 |
| Type of abdominal surgery | | | | 0.042 |
| Previous upper abdominal surgery | 23 (13) | 15 (11) | 8 (23) | |
| Previous lower abdominal surgery | 33 (19) | 26 (19) | 7 (20) | |
| Previous upper and lower abdominal surgery | 13 (7) | 8 (6) | 5 (14) | |
| Diabetes mellitus | 54 (31) | 46 (32) | 8 (23) | 0.27 |
| Hypertension | 54 (31) | 46 (32) | 8 (23) | 0.27 |
| Hyperlipidemia | 32 (18) | 29 (20) | 3 (9) | 0.10 |
| Metabolic syndrome | 39 (22) | 36 (25) | 3 (9) | 0.032 |
| Coronary artery disease | 26 (15) | 20 (14) | 6 (17) | 0.65 |
| Cardiac arrythmia | 38 (21) | 27 (19) | 11 (31) | 0.11 |
| **Pre-LT acuity** | | | | |
| MELD-Na on ICU admission | 38.4 (35.4–41.5) | 38.9 (35.9–41.5) | 37.8 (33.6–41.9) | 0.26 |
| CLIF-C ACLF on ICU admission | 58.5 (54.0–66.8) | 58.4 (53.6–64.1) | 58.2 (52.4–65.9) | 0.79 |
| Number of organ failures at ICU | 3.0 (1.0–4.0) | 2.0 (2.0–3.0) | 3.0 (2.0–4.0) | 0.20 |
| CLIF-C organ failure score at ICU | 13.0 (10.0–15.0) | 13.0 (12.0–14.0) | 13.0 (12.0–15.0) | 0.38 |
| MELD-Na at LT | 41.0 (38.5–42.8) | 41.0 (38.3–43.0) | 40.7 (39.1–42.8) | 0.90 |
| CLIF-C ACLF at LT | 68.0 (62.8–73.0) | 67.8 (62.8–71.9) | 68.7 (65.7–72.5) | 0.34 |
| Number of organ failures at LT | 3.0 (3.0–4.0) | 4.0 (4.0–5.0) | 4.0 (4.0–5.0) | 0.48 |
| CLIF-C organ failure score at LT | 15.0 (14.0–16.0) | 16.0 (14.0–17.0) | 16.0 (15.0–17.0) | 0.29 |
| Base excess at LT | 0.0 (-1.0–2.0) | 1.0 (-1.0–2.0) | 0.0 (-2.0–1.0) | 0.004 |
| **Reason for mechanical ventilation** | | | | 0.011 |
| Lung pathology (pneumonia/ARDS) | 103 (58) | 76 (54) | 27 (77) | |
| Hepatic encephalopathy/airway protection | 74 (42) | 66 (47) | 8 (23) | |
| **Pre-LT sepsis characteristics** | | | | |
| Sepsis within 1 month before LT | 105 (59) | 84 (60) | 21 (60) | 0.96 |
| Interval from sepsis to LT | | | | 0.040 |
| No infection | 39 (22) | 35 (25) | 4 (11) | |
| ≤14 days | 103 (58) | 76 (54) | 27 (77) | |
| >14 days | 35 (20) | 31 (22) | 4 (11) | |
| Infection with MRDO | 17 (10) | 12 (9) | 5 (14) | 0.18 |
| Source of sepsis | | | | |
| Pneumonia | 96 (54) | 74 (52) | 22 (63) | 0.38 |
| SBP | 3 (2) | 3 (2) | 0 (0) | – |
| UTI | 44 (25) | 32 (23) | 12 (34) | 0.24 |
| Other | 12 (7) | 7 (5) | 5 (14) | 0.12 |
| Infection with MRDO | 17 (10) | 12 (9) | 5 (14) | 0.18 |
| **Pre-LT hospitalization characteristics** | | | | |
| Listing date to LT (d) | 18.0 (6.0–157.0) | 18.0 (6.0–146) | 31.0 (7.0–228) | 0.54 |
| Duration of preoperative admission (d) | 21.0 (14.0–31.0) | 21.0 (15.0–32.0) | 17.0 (11.0–29.0) | 0.21 |
| Duration of preoperative ICU admission (d) | 17.0 (9.0–23.0) | 17.0 (9.0–23.0) | 13.0 (9.0–24.0) | 0.74 |
| Duration of preoperative RRT (d) | 12.0 (6.0–20.0) | 13.0 (7.0–21.0) | 9.0 (5.0–12.5) | 0.008 |
| ICU admission before listing | 91 (51) | 74 (52) | 17 (49) | 0.85 |
| OSH admission | 131 (74) | 108 (76) | 23 (66) | 0.21 |
| Duration of OSH admission (d) | 4.0 (0.0–11.0) | 5.0 (0.0–12.0) | 0.0 (0.0–5.0) | 0.07 |
| **Donor and operative characteristics** | | | | |
| Donor risk index | 1.3 (1.1–1.6) | 1.3 (1.1–1.6) | 1.4 (1.2–2.3) | 0.011 |
| DCD donor | 4 (2) | 3 (2) | 1 (9) | 1.00 |
| Cold ischemic time (h) | 7.0 (6.0–8.0) | 7.2 (6.2–8.3) | 7.2 (6.0–8.2) | 0.68 |
| Simultaneous liver–kidney transplant | 14 (8) | 14 (10) | 0 (0) | – |
| PRBC transfusion (u) | 23.0 (16.0–36.0) | 23.0 (15.0–36.0) | 31.0 (18.0–50.0) | 0.049 |
| Venovenous bypass | 122 (69) | 92 (65) | 30 (86) | 0.017 |
| Worst base excess | −8.7 (−11.9 to −6.5) | −8.3 (−11.0 to −6.0) | −9.4 (−13.1 to −7.0) | 0.033 |
| Base excess at end of LT | −2.0 (−4.0–1.1) | −1.3 (−3.6–1.5) | −3.6 (−5.4–0.0) | 0.013 |

Categorical variables are displayed as n (%) and differences were assessed using the $X^2$ test (significant when *p* <0.05). Continuous variables are displayed as median (IQR) and differences were assessed using the Mann–Whitney *U* test (significant when *p* <0.05). ACLF, acute-on-chronic liver failure; ARDS, acute respiratory distress syndrome; CLIF-C, Chronic Liver Failure Consortium; DCD, donation after circulatory death; ICU, intensive care unit; LT, liver transplant; MELD, model for end-stage liver disease; MRDO, multi-drug resistant organism; OSH, outside hospital; PRBC, packed red blood cells; RRT, renal replacement therapy; SBP, spontaneous bacterial peritonitis; UTI, urinary tract infection.

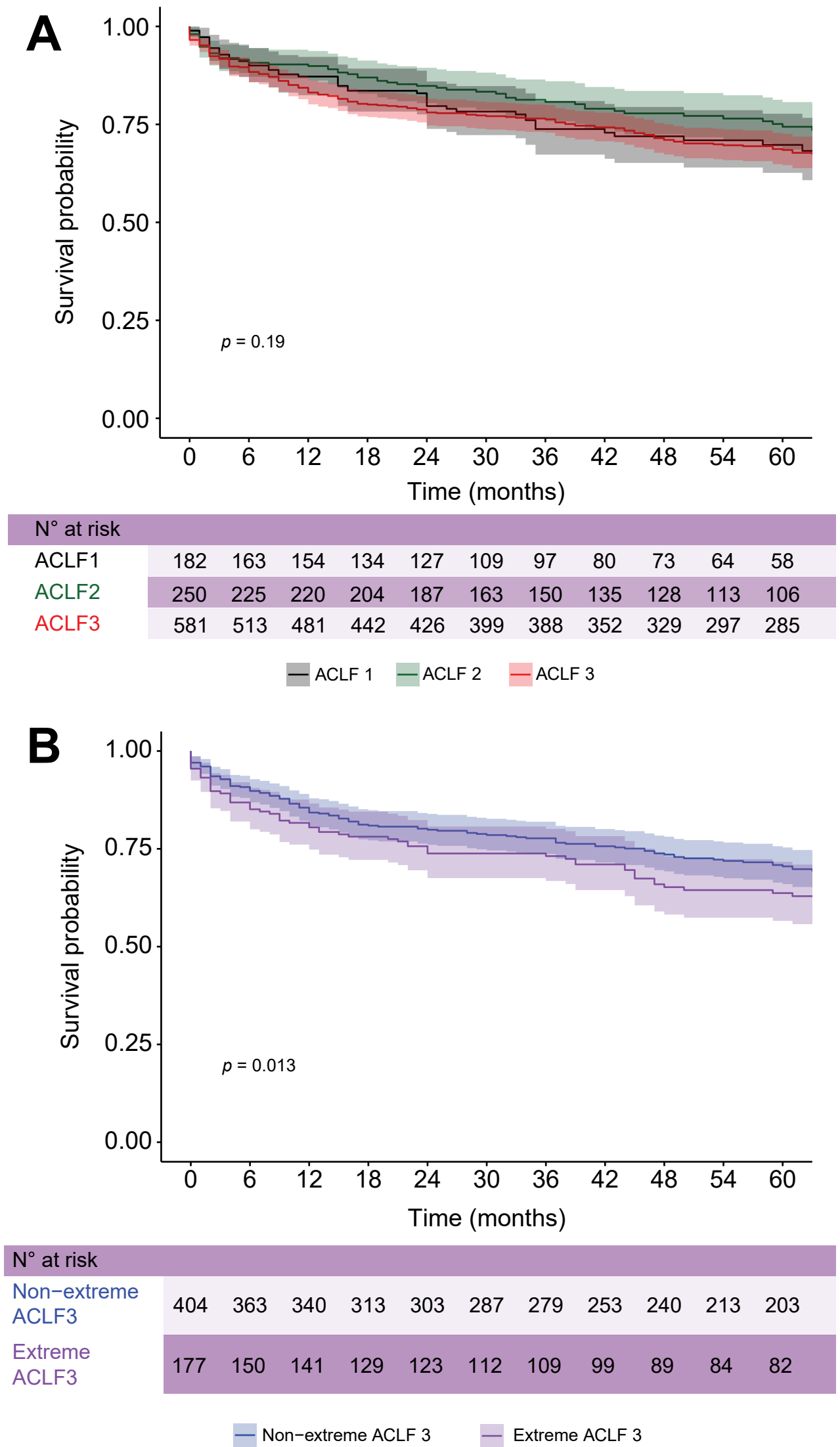


**Fig. 2. Survival comparisons between patients with ACLF 1, 2, and 3, and non-extreme and extreme ACLF 3.** (A) Survival comparisons between patients with ACLF 1, 2, and 3 (Kaplan–Meier method, log-rank test: $p = 0.19$). (B) Survival comparison between patients with non-extreme ACLF 3 and extreme ACLF 3 (Kaplan–Meier method, log-rank test: $p = 0.013$, statistically significant). Shaded area represents 95% confidence intervals.

24%, $p = 0.030$), with a larger proportion of patients experiencing the futile outcome (30% *vs.* 20%, $p = 0.046$). MODEL cohort patients had worse 3-, 6-, and 12-month survivals of 83%, 79%, and 70%, compared with the UCLA cohort with 89%, 85%, and 80%, respectively (Fig. 3A, $p = 0.048$). However, overall survival was equivalent, with the MODEL cohort patients having 1-year, 3-year, and 5-year overall survivals of 70%, 65%, and 59%, compared with 80%, 73%, and 63%, respectively, in the UCLA cohort (Fig. 3B, $p = 0.14$).

## Discussion

In this study, we report the largest single-center experience of ACLF-3 (n = 581) and the only study to define a unique subset of patients with eACLF-3 receiving maximal life support (mechanical ventilation, vasopressors, and renal replacement therapy) at time of LT. Twenty percent of our eACLF-3 cohort experienced a futile outcome, making the overall 5-year survival 63%, which may be considered an unacceptably high short-term mortality to justify the broad use of scarce donor livers. However, considering the high acuity of these patients with eACLF-3, it was remarkable to see that patients with ACLF-3 on maximal life support can achieve acceptable long-term survival with appropriate selection.[28] The 177 patients with eACLF-3 examined at our center had overall consistent patient characteristics and outcomes when compared with the 169 patients in the MODEL Consortium external validation cohort.

As it pertains to this unique population of patients with eACLF-3, significant risk factors for futility in this study included a decrease in $PaO_2$:$FiO_2$ ratio, sepsis diagnosed within 14 days of LT, and the indication for mechanical ventilation. In a 2021 expert consensus panel on futility in LT for patients with ACLF-3, Weiss *et al.*[21] reported their prescribed criteria for futility in ACLF-3 including persistent fever or <72 h of antimicrobial therapy in the case of ongoing sepsis, $PaO_2$:$FiO_2$ ratio <150 at LT, levophed dose >1 µg/kg per min, serum lactate >9 mmol/L. Based on our data, the receipt of antimicrobial therapy for 72 h before LT in the case of sepsis may be inadequate for patients with eACLF-3 on maximal life support, with a longer observation period between sepsis onset and LT allowing for optimal treatment of sepsis and affording the greatest reduction in risk of futility. The decision to proceed with LT in a patient with sepsis must balance the concern about delaying transplant too long, resulting in further decompensation and an inability to perform surgery safely with the risk of infection recrudescence following LT with the addition of immunosuppression. Additionally, the incorporation of acuity measure changes from ICU admission to LT may be better suited to this population with a decrease in $PaO_2$:$FiO_2$ ratio from ICU to LT being more predictive of futility than a specific value cut-off at LT. Given that ACLF is associated with a rapid decompensation leading to organ failure, the rate of change of decompensation may better inform prognosis and the likelihood of futility following LT than acuity measures at LT alone.[29,30]

To our knowledge, this is the first study that specifically analyzed the indication for mechanical ventilation in patients with ACLF-3 as a predictor of post-LT outcomes. Determining indications for intubation can be a difficult task, requiring manual physician chart review with analysis of chest X-rays and culture data. Neither the TAM score nor the SALT-M score included indication for ventilation as a risk factor in the final model and these data are not available in national transplant registries, highlighting the significance of the elucidation of this specific risk factor in this study. Patients with ACLF-3 can require mechanical ventilation for a variety of reasons, and it is not surprising that those intubated for airway protection as a result of severe hepatic encephalopathy or esophageal variceal bleeding with no underlying pulmonary pathology have a lower risk of futility than those intubated for respiratory failure because of pneumonia or ARDS. In fact, among the 35 patients who experienced futility, 29 (83%) of them were intubated for pulmonary pathology preoperatively, with 21 (60%) patients never extubated postoperatively and expiring while intubated. In those futile patients who were extubated postoperatively, eight (23%) were re-intubated before discharge or mortality.

**Table 2. Secondary outcomes in patients with extreme ACLF-3 with comparisons made between the non-futile and futile groups and specific causes of death in the futile group.**

| Variable | Total (N = 177) | Non-futile (n = 142) | Futile (n = 35) | *p* values |
|---|---|---|---|---|
| Post-LT ICU length of stay (days) | 29.0 (14.0–54.0) | 26.0 (15.0–49.0) | 44.0 (10.0–73.0) | 0.41 |
| Post-LT length of stay (days) | 53.0 (32.0–78.5) | 53.0 (33.0–80.0) | 56.0 (14.0–80.0) | 0.65 |
| Time of intubation post-LT | 9.0 (3.0–44.0) | 8.0 (3.0–27.5) | 45.0 (7.0–94.0) | 0.003 |
| Number of reoperations | | | | 0.071 |
| 0 | 76 (43) | 64 (45) | 12 (34) | |
| 1 | 61 (35) | 51 (36) | 10 (29) | |
| ≥2 | 40 (23) | 27 (19) | 13 (37) | |
| Unplanned reoperations | | | | 0.025 |
| 0 | 120 (68) | 103 (73) | 17 (49) | |
| 1 | 34 (19) | 23 (16) | 11 (31.) | |
| ≥2 | 23 (13) | 16 (11) | 7 (20) | |
| Time to first reoperation | | 8.0 (4.0–16.0) | 5.0 (2.0–12.0) | 0.25 |
| Postoperative respiratory failure | 118 (67) | 84 (59) | 34 (97) | <0.001 |
| Postoperative neurological failure | 33 (19) | 12 (9) | 21 (60) | <0.001 |
| Postoperative cardiac failure | 38 (22) | 14 (10) | 24 (69) | <0.001 |
| Postoperative sepsis | 95 (54) | 76 (54) | 19 (54) | 0.013 |
| Portal vein thrombosis | 2 (1) | 1 (1) | 1 (3) | 0.28 |
| Hepatic artery thrombosis | 9 (5) | 5 (4) | 4 (11) | 0.057 |
| Primary graft non-function | 6 (3) | 3 (2) | 3 (9) | 0.171 |
| Discharge to SNF | 10 (6) | 8 (6) | 2 (6) | <0.001 |
| Discharge to LTACH | 11 (6) | 11 (8) | 0 (0) | <0.001 |
| Post-LT readmission within 1 month | 43 (24) | 39 (28) | 4 (11) | 0.046 |
| Post-LT dialysis dependence | 22 (12) | 16 (11) | 6 (17) | 0.35 |
| Post-LT tracheostomy | 47 (27) | 34 (24) | 13 (37) | 0.11 |
| Re-LT | 10 (6) | 8 (6) | 2 (6) | 0.57 |
| Discharged following index LT | – | – | 9 (26) | – |
| Cause of death | | | | |
| Cerebral hemorrhage | – | – | 3 (9) | – |
| HAT | – | – | 2 (6) | – |
| MSOF – no sepsis | – | – | 3 (9) | – |
| PE | – | – | 1 (3) | – |
| Pneumonia with sepsis, MSOF | – | – | 3 (9) | – |
| PNF | – | – | 3 (9) | – |
| Delayed graft function with MSOF | | | 3 (9) | |
| Sepsis with MSOF | – | – | 8 (23) | – |
| Other | | | 6 (17) | |
| Unknown | – | – | 3 (9) | – |

Categorical variables are displayed as n (%) and differences were assessed using the $X^2$ test (significant when $p$ <0.05). Continuous variables are displayed as median (IQR) and differences were assessed using the Mann–Whitney *U* test (significant when $p$ <0.05). ACLF, acute-on-chronic liver failure; HAT, hepatic artery thrombosis; ICU, intensive care unit; LT, liver transplant; LTACH, long-term acute care hospital; MSOF, multisystem organ failure; PE, pulmonary embolism; PNF, primary non-function; SNF, skilled nursing facility.

In addition to indication for mechanical ventilation, worsening base deficit at LT was identified as a significant risk factor for futility, which is consistent with prior reports of increasing lactate level (another marker of tissue ischemia and systemic inflammation) being associated with futility in patients with ACLF-3.[21,24] Lactate was not routinely collected at all time points at our center so we could not assess its impact on futility. Given that nearly 35% of the futile patients had multisystem organ failure as a cause of death, base deficit may be an important indicator of unrecoverable global tissue ischemia that portends futile outcomes even with LT. Additionally, within the 35% who died as a result of multisystem organ failure, 29% had associated sepsis, again signifying the prognostic importance of sepsis in this population. Shorter duration of

**Table 3. Results of multivariate logistic regression modeling futility.**

| Variable | Risk score points | β-coefficients | OR | 95% CI | *p* values |
|---|---|---|---|---|---|
| Intercept | 0 | −4.0 | | | |
| Previous upper abdominal surgery | 6 | 1.5 | 4.6 | 1.8–12.1 | 0.002 |
| Sepsis duration (ICU to LT) | | | | | |
| None | 0 | – | – | – | – |
| ≤14 days | 4 | 1.8 | 5.9 | 1.5–24.1 | 0.013 |
| >14 days | 1 | 1.0 | 2.6 | 0.7–9.6 | 0.14 |
| Intubation indication | | | | | |
| Airway protection | 0 | – | – | – | – |
| Pneumonia/ARDS | 6 | 1.3 | 3.5 | 1.4–9.2 | 0.010 |
| Decrease in $PaO_2$:$FiO_2$ ≥12% per week from ICU to LT | 7 | 1.4 | 3.9 | 1.4–10.6 | 0.008 |
| Base deficit at LT | 5 | 0.2 | 1.3 | 1.1–1.5 | 0.007 |

Displayed $p$ values are determined using the Wald test, $p$ <0.05 was considered significant. ARDS, acute respiratory distress syndrome; ICU, intensive care unit; LT, liver transplant; OR, odds ratio.

**Table 4. Baseline demographic and outcomes comparison in patients with extreme ACLF-3 at UCLA and external MODEL Consortium cohort.**

| Variable | UCLA (n = 177) | MODEL (n = 169) | *p* values |
|---|---|---|---|
| **Demographic** | | | |
| Age (years) | 58.00 [49.6, 63.9] | 55.00 [46.0, 61.3] | 0.016 |
| Sex = M | 89 (50) | 78 (46.2) | 0.51 |
| BMI (kg/m$^2$) | 26.6 [23.6, 31.7] | 29.9 [24.9, 36.0] | 0.001 |
| Primary diagnosis | | | 0.39 |
| Alcohol | 65 (37) | 65 (39) | |
| MASLD | 45 (25) | 27 (16) | |
| Hepatitis C | 34 (19) | 29 (17) | |
| Autoimmune/cholestasis | 21 (12) | 25 (15) | |
| Other | 12 (7) | 23 (14) | |
| Prior abdominal surgery | 70 (40) | 70 (42) | 0.77 |
| Diabetes mellitus | 54 (31) | 40 (24) | 0.20 |
| **Pre-LT acuity/hospitalization characteristics** | | | |
| MELD-Na on ICU admission | 38.4 [35.4, 41.5] | 36.0 [32.0, 40.0] | <0.001 |
| MELD-Na at LT | 41.0 [38.5, 42.8] | 37.7 [34.8, 41.3] | <0.001 |
| Reason for mechanical ventilation | | | 0.009 |
| Lung pathology (pneumonia/ARDS) | 103 (58) | 71 (43) | |
| Hepatic encephalopathy/airway protection | 74 (42) | 98 (58) | |
| $PaO_2$:$FiO_2$ ratio at ICU admission | 247.1 [165.0, 334.2] | 255.0 [161.6, 349.3] | 0.92 |
| $PaO_2$:$FiO_2$ ratio at LT | 303.3 [230.0, 387.5] | 259.0 [211.4, 330.6] | 0.003 |
| Preoperative sepsis | 105 (60) | 96 (57) | 0.66 |
| Listing date to LT (days) | 18.0 [6.0, 157.0] | 7.0 [3.0, 43.5] | <0.001 |
| Duration of preoperative ICU admission (days) | 17.0 [9.0, 23.0] | 9.0 [5.0, 16.0] | <0.001 |
| **Donor and operative characteristics** | | | |
| Donor age (years) | 34.0 [22.0, 49.0] | 35.5 [26.0, 49.8] | 0.14 |
| Cold ischemic time (h) | 7.0 [6.0, 8.0] | 5.9 [4.4, 7.9] | 0.001 |
| DCD | 4 (2) | 18 (11) | 0.003 |
| **Outcomes** | | | |
| Post-LT ICU length of stay (days) | 29.0 [14.0, 54.0] | 9.0 [4.0, 18.0] | <0.001 |
| Post-LT length of stay (days) | 53.0 [32.0, 78.5] | 22.0 [14.0, 36.0] | <0.001 |
| Post-LT duration of intubation | 9.0 [3.0, 44.0] | 2.0 [1.0, 5.3] | <0.001 |
| Extubated after LT | 20 (11) | 7 (4) | 0.25 |
| PNF | 6 (3) | 5 (3) | 0.81 |
| HAT | 9 (5) | 2 (1) | 0.08 |
| Discharge location | | | 0.007 |
| Home | 76 (53) | 92 (63) | |
| LTAC | 11 (6) | 20 (14) | |
| Rehab or SNF | 57 (40) | 35 (24) | |
| Post-LT readmission within 1 month | 43 (24) | 60 (36) | 0.030 |
| Biliary complications | 29 (17) | 30 (18) | 0.84 |
| Re-LT | 10 (6) | 1 (1) | 0.017 |
| Futility | 35 (20) | 50 (30) | 0.046 |

Categorical variables are displayed as n (%) and differences were assessed using the $X^2$ test (significant when $p$ <0.05). Continuous variables are displayed as median (IQR) and differences were assessed using the Mann–Whitney *U* test (significant when $p$ <0.05). ACLF, acute-on-chronic liver failure; ARDS, acute respiratory distress syndrome; CLIF-C, Chronic Liver Failure Consortium; DCD, donation after circulatory death; ICU, intensive care unit; LT, liver transplant; MASLD, metabolic dysfunction-associated steatotic liver disease; MELD, model for end-stage liver disease; MODEL, Multi-Organ Dysfunction and Evaluation for Liver Transplantation; OSH, outside hospital; SNF, skilled nursing facility.

sepsis with worsening base deficit may suggest overwhelming sepsis leading to multisystem organ failure that cannot be salvaged even with LT.

An increasing donor risk index was correlated with futility in demographic comparisons, validating that high-risk donor livers are ill suited to such critically ill patients with ACLF-3 on maximal life support.[31,32] In fact, in our cohort only four patients with eACLF-3 received donation after circulatory death (DCD) livers, showing that our institutional practice has been to prioritize higher quality organs for the most significantly ill recipients. However, in the current era of machine perfusion, the need to avoid organs traditionally considered marginal for the highest acuity recipients may not be as relevant because of the afforded ability to mitigate ischemia–reperfusion injury and assess organ function before transplantation with normothermic machine perfusion. Going forward, whether it is suitable to increasingly utilize DCD livers for these high-acuity recipients in the era of widespread machine perfusion will certainly be an area of active investigation.[33]

Our multivariate logistic regression model predicted futility in patients with eACLF-3 with an AUROC of 0.74 (SD 0.15), incorporating dynamic changes in oxygenation status along with the novel risk factor of pulmonary pathology as an indication for intubation. We chose to select a decision threshold at a PPV ≥50% in each training set as this corresponds to ∼50% of patients above the threshold experiencing futility. This threshold is associated with a risk score value of 8 and would allow a relatively high specificity and minimization of false positive results with a relatively lower sensitivity (sensitivity: 0.57, specificity 0.81, and PPV 0.45). In the context of selecting patients with eACLF-3 for LT, a relatively lower number of incorrect futility predictions would be desired to not exclude consideration of a patient for receiving a potentially lifesaving LT. Given the relatively low number of events in our

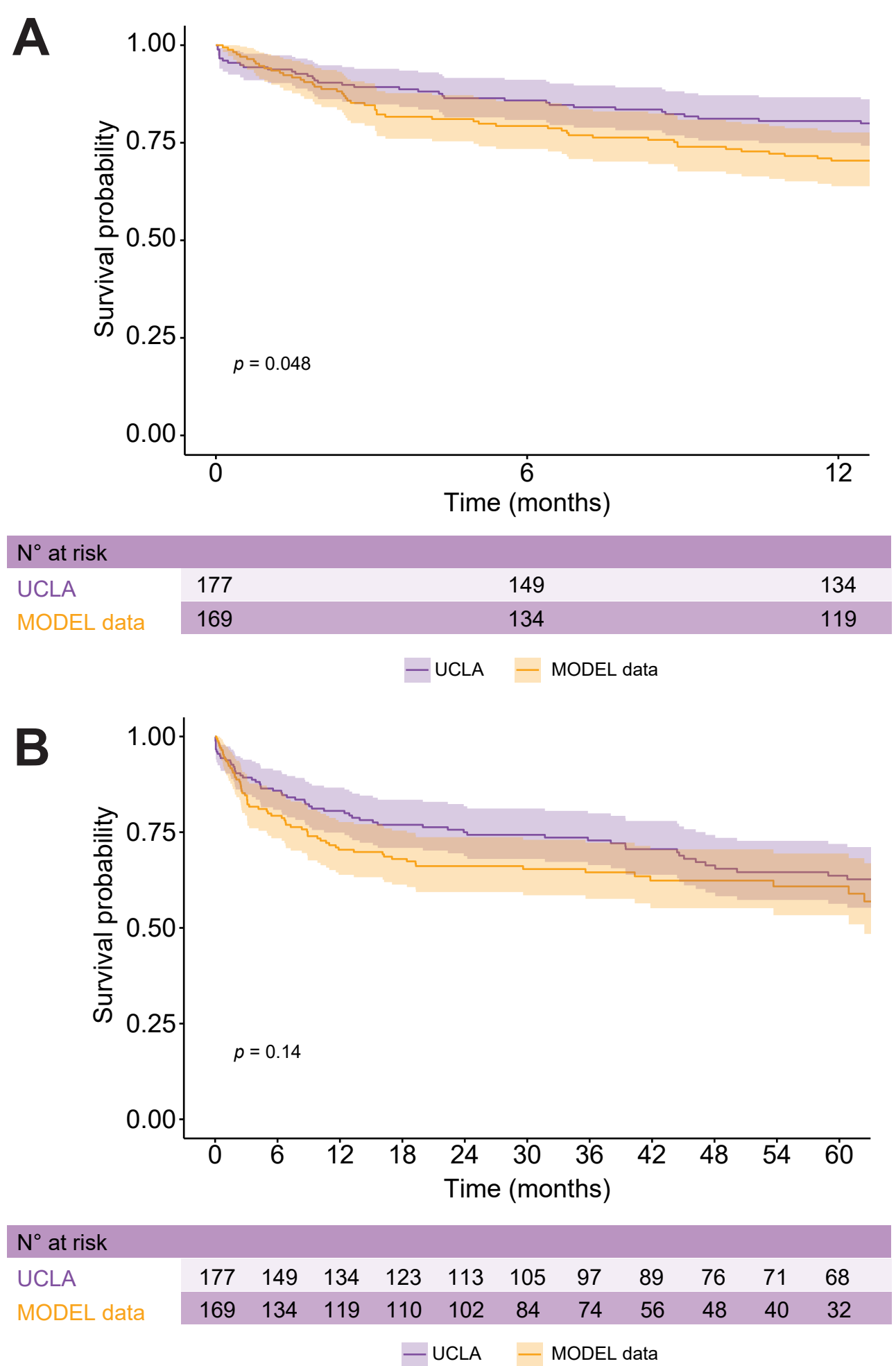


**Fig. 3. One-year and overall survival comparisons between patients with extreme ACLF-3 at UCLA (purple) and in the MODEL Consortium (orange).** Survival differences were assessed using the Kaplan–Meier method and log-rank test (A: $p$ = 0.048, statistically significant; B: $p$ = 0.14). Shaded area represents 95% confidence intervals.

cohort, our current model may be overfit to the training data, and a degree of instability is demonstrated by large standard deviations associated with evaluation metrics from cross-validation. Validation in an external dataset is required prior to clinical adoption to predict futility in this population. Although we were able to obtain the MODEL Consortium external validation data, the granularity of data necessary to validate our model was not present. However, key takeaways from these data are that patients with eACLF-3 exist in the LT population with broadly similar characteristics to our cohort, although are rare (n = 169 in 15 centers). Additionally, a similar survival trend was seen where the vast majority of mortality is seen in the short term, with futile outcomes in eACLF-3 observed in 20% of the UCLA cohort and 30% of the MODEL cohort. However, patients with eACLF-3 surviving that first year have acceptable long-term survival, emphasizing that with appropriate patient selection these patients can be transplanted successfully.

### Limitations

Because of the retrospective design of this study, we cannot account for all potential confounding variables, preventing determination of any causal relationships. We focused on patients with eACLF-3 who were transplanted and analyzed risk factors, although we must acknowledge that by design this presents a possible selection bias, whereby the specific subset of listed patients with eACLF-3 surviving to and following transplant differed from patients with eACFL-3 experiencing pre-LT mortality. There were ∼285 patients with eACLF-3 listed for transplant at our center, 108 (38%) of which experienced waitlist dropout without transplant. Additionally, our findings regarding post-transplant survival were broadly corroborated in an external validation cohort. Although the model itself cannot be externally validated because of missing data in the MODEL Consortium data, it should be noted that the main contribution of our manuscript is a description of outcomes in this unique subset of eACLF-3 recipients on maximal pre-LT life support. Our model certainly adds insight into some of the factors that may portend a poor outcome in this subset of patients but will require additional prospective data for validation and testing moving forward. Although our center experience with transplantation of many patients with ACLF-3 and eACLF-3 may not be directly generalizable to other centers with lower acuity patient populations, the similar outcomes observed in the eACFL-3 MODEL population support that acceptable outcomes are achievable with careful patient selection. Futility in LT is not clearly defined and can have differing meanings depending on context.[34] In this work, we defined futility as 1-year post-transplant death or in-hospital mortality, which is appropriate when considering transplantation for a hyperacute population.

## Conclusions

We report the largest single-center experience of LT for patients with ACLF-3, and define a unique hyperacute cohort of patients with eACLF-3 all of whom required mechanical ventilation, vasopressors, and dialysis before LT. In this subset of patients with eACLF-3, we demonstrate excellent short- and long-term survival considering the high acuity of these patients, which is validated in an external cohort of patients from the MODEL Consortium. We propose novel risk factors associated with futility in this population which may aid in appropriate selection of patients with eACLF-3 requiring maximal life support for lifesaving LT.

**Affiliations**

[20]*Departement Hospitalo-Universitaire, Hepatinov, Villejuif, France;* [1]*Dumont-UCLA Transplant and Liver Cancer Centers, Department of Surgery, David Geffen School of Medicine at University of California, Los Angeles, CA, USA;* [2]*Section of Gastroenterology, Michael E. DeBakey Veterans Affairs Medical Center, Houston, TX, USA;* [3]*VA HSR&D Center for Innovations in Quality, Effectiveness and Safety, Michael E. DeBakey VA Medical Center, Houston, TX, USA;* [4]*Section of Gastroenterology and Hepatology, Department of Medicine, Baylor College of Medicine, Houston, TX, USA;* [5]*Department of Critical Care Medicine and Division of Gastroenterology (Liver Unit), University of Alberta, Edmonton, AB, Canada;* [6]*Division of Gastrointestinal & Liver Disease, Keck Hospital at University of Southern California, Los Angeles, CA, USA;* [7]*Piedmont Transplant Institute, Piedmont Atlanta Hospital, Atlanta, GA, USA;* [8]*Division of Gastroenterology & Hepatology, University of Maryland School of Medicine, Baltimore, MD, USA;* [9]*Department of Gastroenterology, Hepatology and Nutrition, Cleveland Clinic, Cleveland, OH, USA;* [10]*Division of Gastroenterology*

*and Hepatology, Department of Medicine, Northwestern University, Chicago, IL, USA; [11]Division of Gastroenterology and Hepatology, Mayo Clinic, Rochester, MN, USA; [12]Baylor University Medical Center, Division of Hepatology. Baylor Scott and White Hospital, Dallas, TX, USA; [13]Division of Gastroenterology, Hepatology and Nutrition, Vanderbilt University, Nashville, TN, USA; [14]Department of Medicine, University of Colorado, Aurora, CO, USA; [15]Recanati/Miller Transplantation Institute, Icahn School of Medicine at Mount Sinai, New York, NY, USA; [16]Division of Transplant and Hepatobiliary Diseases, Department of Surgery, Lahey Hospital and Medical Center, Burlington, MA, USA; [17]Medicine, Division of Gastroenterology, Oregon Health and Science University, Portland, OR, USA; [18]Division of Hepatology, Department of Medicine, Montefiore Medical Center, Bronx, NY, USA; [19]Karsh Division of Gastroenterology and Hepatology, Comprehensive Transplant Center, Cedars-Sinai Medical Center, Los Angeles, CA, USA*

## Abbreviations

ACLF, acute-on-chronic liver failure; AIC, Akaike information criterion; ARDS, acute respiratory distress syndrome; CANONIC, Consortium Acute-on-chronic Liver Failure in Cirrhosis; CLIF-C, Chronic Liver Failure Consortium; DCD, donation after circulatory death; eACLF-3, extreme ACLF grade 3; HAT, hepatic artery thrombosis; ICU, intensive care unit; LT, liver transplant; LTACH, long-term acute care hospital; MELD, model for end-stage liver disease; MODEL, Multi-Organ Dysfunction and Evaluation for Liver Transplantation Consortium; MRDO, multidrug resistant organism; MSOF, multisystem organ failure; OR, odds ratio; OSH, outside hospital; $PaO_2$:$FiO_2$, partial pressure of oxygen in arterial blood to the fraction of inspiratory oxygen concentration ratio; PE, pulmonary embolism; PNF, primary non-function; PPV, positive predictive value; PRBC, packed red blood cells; RRT, renal replacement therapy; SALT-M, Sundaram ACLF-LT-Mortality; SBP, spontaneous bacterial peritonitis; SNF, skilled nursing facility; TAM, transplantation for ACLF-3 Model; UCLA, University of California, Los Angeles; UNOS, united network for organ sharing; UTI, urinary tract infection.

## Financial support

RH is a core investigator at the Center for Innovations in Quality, Effectiveness and Safety (CIN 13-413), Michael E. DeBakey VA Medical Center, Houston, TX, USA. KS is supported by the National Institutes of Health (U01CA230690).

## Conflicts of interest

The authors have no conflicts of interest related to this work.

Please refer to the accompanying ICMJE disclosure forms for further details.

## Authors' contributions

Participated in the study concept and design, acquisition of data, interpretation of data, and supervision: all authors. Data collection, analysis, and manuscript writing: AM, AT, DM. The Consortium authors listed at the end of this manuscript were essential collaborators in the acquisition of the multicenter MODEL Consortium data.

## Data availability

The de-identified data are only internally available for the MODEL Consortium researchers after approval of the Ancillary Studies Steering Committee. Single-center data may be available.

## Acknowledgements

We would like to thank the authors from the MODEL Consortium for their assistance in collecting the critical external validation data.

## Supplementary data

Supplementary data to this article can be found online at https://doi.org/10.1016/j.jhepr.2026.101959.

## Additional authors from the Multi-Organ Dysfunction and Evaluation for Liver Transplantation (MODEL Consortium)

Atef Al Attar[18], Kambiz Kosari[18], Richard Garcia (Departement Hospitalo-Universitaire, Hepatinov, Villejuif, France), Gevork Salmastyan (Departement Hospitalo-Universitaire, Hepatinov, Villejuif, France), William Cranford[6], Preet Patel[7], Pei Xue[7], Soumya Mishra[7], Madison Parks[7], Gianina Flocco[8], Jing Gao[9], Tiffany Wu[10], Priya Thanneeru[10], Vikrant Reddy[11], Jing Gao[11], Mariana Hurtado[11], Islam Mohamed[4], Ross Vyhmeister[12], Christine R Lopez[12], Braidie Campbell[13], Adam C. Winters[14], Mary Ann Simpson[15], Xiaohan Ying (Department of Medicine, Weill Cornell Medicine, New York, NY, USA).

## References

*Author names in bold designate shared co-first authorship*

[1] Moreau R, Jalan R, Gines P, et al. Acute-on-chronic liver failure is a distinct syndrome that develops in patients with acute decompensation of cirrhosis. Gastroenterology 2013;144:1426–1437, 37.e1–9.
[2] Piano S, Mahmud N, Caraceni P, et al. Mechanisms and treatment approaches for ACLF. Liver Int 2025;45:e15733.
[3] Gustot T, Fernandez J, Garcia E, et al. Clinical course of acute-on-chronic liver failure syndrome and effects on prognosis. Hepatology 2015;62:243–252.
[4] Moreau R, Gao B, Papp M, et al. Acute-on-chronic liver failure: a distinct clinical syndrome. J Hepatol 2021;75:S27–S35.
[5] Arroyo V, Moreau R. Diagnosis and prognosis of acute on chronic liver failure (ACLF) in cirrhosis. J Hepatol 2017;66:451–453.
[6] Jalan R, Gines P, Olson JC, et al. Acute-on chronic liver failure. J Hepatol 2012;57:1336–1348.
[7] Queck A, Weiler N, Trebicka J. Transplantation in acute-on-chronic liver failure: feasibility and futility. Clin Liver Dis 2022;19:191–193.
[8] Trebicka J, Gu W, Ibáñez-Samaniego L, et al. Rebleeding and mortality risk are increased by ACLF but reduced by pre-emptive TIPS. J Hepatol 2020;73:1082–1091.
[9] Trebicka J, Fernandez J, Papp M, et al. The PREDICT study uncovers three clinical courses of acutely decompensated cirrhosis that have distinct pathophysiology. J Hepatol 2020;73:842–854.
[10] Jalan R, Saliba F, Pavesi M, et al. Development and validation of a prognostic score to predict mortality in patients with acute-on-chronic liver failure. J Hepatol 2014;61:1038–1047.
[11] Arroyo V, Moreau R, Jalan R, et al. ESIL-CLIF Consortium CANONIC Study. Acute-on-chronic liver failure: a new syndrome that will re-classify cirrhosis. J Hepatol 2015;62:S131–S143.
[12] Sundaram V, Shah P, Wong RJ, et al. Patients with acute on chronic liver failure grade 3 have greater 14-day waitlist mortality than status-1a patients. Hepatology 2019;70:334–345.
[13] Durand F, Roux O, Weiss E, et al. Acute-on-chronic liver failure: where do we stand? Liver Int 2021;41:128–136.
[14] Gustot T, Agarwal B. Selected patients with acute-on-chronic liver failure grade 3 are not too sick to be considered for liver transplantation. J Hepatol 2017;67:667–668.
[15] Artru F, Louvet A, Ruiz I, et al. Liver transplantation in the most severely ill cirrhotic patients: a multicenter study in acute-on-chronic liver failure grade 3. J Hepatol 2017;67:708–715.
[16] Artru F, Goldberg D, Kamath PS. Should patients with acute-on-chronic liver failure grade 3 receive higher priority for liver transplantation? J Hepatol 2023;78:1118–1123.
[17] Michard B, Artzner T, Deridder M, et al. Pretransplant intensive care unit management and selection of grade 3 acute-on-chronic liver failure transplant candidates. Liver Transplant 2022;28:17–26.
[18] Sundaram V, Patel S, Shetty K, et al. Risk factors for posttransplantation mortality in recipients with grade 3 acute-on-chronic liver failure: analysis of a North American consortium. Liver Transplant 2022;28:1078–1089.
[19] Wu T, Sundaram V. Transplantation for acute-on-chronic liver failure. Clin Liver Dis 2019;14:152–155.
[20] Michard B, Artzner T, Lebas B, et al. Liver transplantation in critically ill patients: preoperative predictive factors of post-transplant mortality to avoid futility. Clin Transplant 2017;31:e13115.
[21] Weiss E, Saner F, Asrani SK, et al. When is a critically ill cirrhotic patient too sick to transplant? Development of consensus criteria by a multidisciplinary panel of 35 international experts. Transplantation 2021;105:561–568.
[22] Engelmann C, Thomsen KL, Zakeri N, et al. Validation of CLIF-C ACLF score to define a threshold for futility of intensive care support for patients with acute-on-chronic liver failure. Critical Care 2018;22:254.

[23] Figorilli F, Putignano A, Roux O, et al. Development of an organ failure score in acute liver failure for transplant selection and identification of patients at high risk of futility. PLoS One 2017;12:e0188151.
[24] Artzner T, Michard B, Weiss E, et al. Liver transplantation for critically ill cirrhotic patients: stratifying utility based on pretransplant factors. Am J Transplant 2020;20:2437–2448.
[25] Hernaez R, Karvellas CJ, Liu Y, et al. The novel SALT-M score predicts 1-year post-transplant mortality in patients with severe acute-on-chronic liver failure. J Hepatol 2023;79:717–727.
[26] Sundaram V, Kogachi S, Wong RJ, et al. Effect of the clinical course of acute-on-chronic liver failure prior to liver transplantation on post-transplant survival. J Hepatol 2020;72:481–488.
[27] Azur MJ, Stuart EA, Frangakis C, et al. Multiple imputation by chained equations: what is it and how does it work? Int J Methods Psychiatr Res 2011;20:40–49.
[28] Kwong AJ, Ebel NH, Kim WR, et al. OPTN/SRTR 2021 annual data report: liver. Am J Transplant 2023;23:S178–S263.
[29] Györi GP, Silberhumer GR, Rahmel A, et al. Impact of dynamic changes in MELD score on survival after liver transplantation–a Eurotransplant registry analysis. Liver Int 2016;36:1011–1017.
[30] Cholankeril G, Li AA, Dennis BB, et al. Pre-operative delta-MELD is an independent predictor of higher mortality following liver transplantation. Sci Rep 2019;9:8312.
[31] Nemes B, Gelley F, Zádori G, et al., editors. Outcome of liver transplantation based on donor graft quality and recipient status. Transplant Proc 2010;42:2327–2330.
[32] Winter A, Féray C, Antoine C, et al. Matching graft quality to recipient's disease severity based on the survival benefit in liver transplantation. Sci Rep 2020;10:4111.
[33] Parente A, Wehrle CJ, Schold JD, et al. Frequent use of machine perfusion enables utilization of higher-risk DCD livers in the United States: are we there yet? J Hepatol 2024;81. https://doi.org/10.1016/j.jhep.2024.06.021.
[34] Rolak S, Elhawary A, Diwan T, et al. Futility and poor outcomes are not the same thing: a clinical perspective of refined outcomes definitions in liver transplantation. Liver Transplant 2024;30:421–430.

Keywords: Acute-on-chronic liver failure; Liver transplant; Maximal life support.
*Received 18 June 2025; received in revised form 30 June 2026; accepted 9 July 2026; Available online 5 August 2026*

Journal of Hepatology, Volume *8*

## Supplemental information

# Liver transplantation at the extreme: Analysis of outcomes and predictors of futility in patients with acute-on-chronic liver failure grade 3 on maximal life support

**Andrew Melehy, Asara Thepbunchonchai, Benjamin V. Tran, Ruben Hernaez, Dominic Amara, Samer Ebaid, Daniela Markovic, Fady M. Kaldas, Igor Barjaktarevic, Victor Xia, Constantine J. Karvellas, Saro Khemichian, Lance L. Stein, Kirti Shetty, Christina C. Lindenmeyer, Justin R. Boike, Douglas A. Simonetto, Robert S. Rahimi, Prasun K. Jalal, Manhal Izzy, Michael S. Kriss, Gene Y. Im, Ming V. Lin, Janice H. Jou, Brett E. Fortune, George Cholankeril, Alexander Kuo, Vinay Sundaram, for the Multi-Organ Dysfunction and Evaluation for Liver Transplantation (MODEL) Consortium, Ronald W. Busuttil, Douglas G. Farmer, and Vatche G. Agopian**

# Liver transplantation at the extreme: analysis of outcomes and predictors of futility in patients with acute-on-chronic liver failure grade 3 on maximal life support

Andrew Melehy, Asara Thepbunchonchai, Benjamin V. Tran, Ruben Hernaez, Dominic Amara, Samer Ebaid, Daniela Markovic, Fady M. Kaldas, Igor Barjaktarevic, Victor Xia, Constantine J. Karvellas, Saro Khemichian, Lance L. Stein, Kirti Shetty, Christina C. Lindenmeyer, Justin R. Boike, Douglas A. Simonetto, Robert S. Rahimi, Prasun K. Jalal, Manhal Izzy, Michael S. Kriss, Gene Y. Im, Ming V. Lin, Janice H. Jou, Brett E. Fortune, George Cholankeril, Alexander Kuo, Vinay Sundaram, for the Multi-Organ Dysfunction and Evaluation for Liver Transplantation (MODEL) Consortium, Ronald W. Busuttil, Douglas G. Farmer, and Vatche G. Agopian

Table of contents

Table S1……………………………………………………………..……………………..2

Table S2…………………………………………………………………………..………..3

Table S3……………………………………………………………..……………………..4

Table S4………………………………………..…………………...……..………...5

Table S5……………………………………………………………...………...………...6

Fig. S1……………………………………………………………...…………………..10

BioRender Publication License……………………………………..……………........11

**Table S1**: Baseline demographic comparisons between extreme and non-extreme ACLF-3 patients

| Variable | Total (n=581) | Non-extreme (n=404) | Extreme (n=177) | P |
|---|---|---|---|---|
| **Demographic** | | | | |
| Age (y), median (IQR) | 57.0 (50.0-63.0) | 57.0 (50.0-62.3) | 58.0 (49.6-63.9) | 0.86 |
| Gender = M (%) | 325 (56) | 236 (58) | 89 (50) | 0.06 |
| BMI (kg/m$^2$), median (IQR) | 27.4 (23.7-31.8) | 27.7 (23.8-31.9) | 26.6 (23.6-31.7) | 0.19 |
| Primary Diagnosis (%) | | | | 0.24 |
| Alcohol | 220 (38) | 155 (38) | 65 (37) | |
| MAFLD | 115 (20) | 70 (17) | 45 (25) | |
| Hepatitis C | 89 (15) | 55 (14) | 34 (19) | |
| Autoimmune/ cholestasis | 54 (9) | 33 (8) | 21 (12) | |
| Other | 103 (18) | 91 (23) | 12 (7) | |
| Previous abdominal surgery | 200 (34) | 144 (36) | 56 (32) | 0.49 |
| Diabetes mellitus | 163 (28) | 109 (27) | 54 (31) | 0.16 |
| **Pre-LT Acuity** | | | | |
| MELD-Na at LT, median (IQR) | 40.0 (37.0-40.0) | 40.0 (38.0-40.0) | 40.0 (37.0-40.0) | 0.35 |
| Mechanical Ventilation at LT, n (%) | 313 (54) | 136 (38) | 177 (100) | <0.001 |
| Dialysis at LT, n (%) | 462 (80) | 285 (71) | 177 (100) | <0.001 |
| **Pre-LT Hospitalization Characteristics** | | | | |
| Listing date to LT (d), median (IQR) | 17.0 (6.0-127.0) | 16.0 (6.0-124.8) | 18.0 (6.0-157.0) | 0.70 |
| Duration of preoperative admission (d), median | 17.0 (10.0-28.0) | 16.0 (9.0-25.0) | 21.0 (14.0-31.0) | <0.001 |
| ICU admission at LT, n (%) | 462 (80) | 285 (71) | 177 (100) | <0.001 |
| **Donor and Operative Characteristics** | | | | |
| Donor Risk Index, median (IQR) | 1.3 (1.1-1.6) | 1.3 (1.1-1.6) | 1.3 (1.1-1.6) | 0.26 |
| DCD donor | 19 (3) | 15 (4) | 4 (2) | 0.77 |
| Cold ischemic time (h), median (IQR) | 7.0 (6.0-8.3) | 7.2 (6.0-8.4) | 7.0 (6.0-8.0) | 0.57 |

ACLF, acute-on-chronic liver failure; IQR, interquartile range; BMI, body mass index; MAFLD, metabolic-associated fatty liver disease; LT, liver transplant; MELD, Model for End-stage Liver Disease; ICU, intensive care unit; DCD, donation after circulatory death.

**Table S2**: Annual center transplant volume and ACLF-3 transplant volume over time with median MELD at transplant in our center and our center's DSA

| Year | Number of liver transplants | Number (%) of ACLF-3 transplants | Number (%) of ACLF-3 transplants for hepatitis C | Median MELD at transplant in southern California donor service area | Median MELD at transplant at our center |
|---|---|---|---|---|---|
| 2010 | 164 | 57 (35) | 10 (18) | 28 | 31 |
| 2011 | 180 | 66 (37) | 19 (29) | 28 | 33 |
| 2012 | 144 | 40 (28) | 10 (25) | 27 | 27 |
| 2013 | 159 | 51 (32) | 10 (20) | 30 | 30 |
| 2014 | 155 | 55 (35) | 9 (16) | 30 | 33 |
| 2015 | 139 | 56 (40) | 8 (14) | 31 | 34 |
| 2016 | 151 | 62 (41) | 4 (6) | 34 | 37 |
| 2017 | 135 | 40 (30) | 4 (10) | 30 | 33 |
| 2018 | 153 | 49 (32) | 0 (0) | 30 | 35 |
| 2019 | 157 | 42 (27) | 0 (0) | 23 | 33 |
| 2020 | 158 | 37 (23) | 4 (11) | 28 | 32 |
| 2021 | 136 | 26 (19) | 1 (4) | 29 | 33 |

ACLF, acute-on-chronic liver failure; MELD, model for end-stage liver disease; DSA, donor service area

| **Table S3:** Univariable Odds Ratios of Categorical Variables | | | | |
|---|---|---|---|---|
| Variable | OR | Lower 95% CI | Upper 95% CI | P |
| | . | . | . | . |
| HCV | 6.649 | 1.330 | 33.236 | 0.021 |
| NASH/Cryptogenic/HBV | 1.750 | 0.331 | 9.250 | 0.51 |
| EtOH | 1.935 | 0.393 | 9.534 | 0.42 |
| Cholestatic/AIH | 1.000 | . | . | . |
| Female | 1.089 | 0.520 | 2.283 | 0.82 |
| Male | 1.000 | . | . | . |
| Previous Lower Abdominal Surgery | 1.633 | 0.602 | 4.429 | 0.34 |
| Previous Upper Abdominal Surgery | 3.429 | 1.433 | 8.203 | 0.006 |
| No Prior Abdominal Surgery | 1.000 | . | . | . |
| Diabetes | 0.618 | 0.261 | 1.467 | 0.28 |
| Hyperlipidemia | 0.365 | 0.104 | 1.277 | 0.12 |
| Metabolic Syndrome | 0.276 | 0.080 | 0.956 | 0.042 |
| Coronary Artery Syndrome | 1.262 | 0.465 | 3.424 | 0.65 |
| Sepsis within 1 month | 1.018 | 0.478 | 2.166 | 0.96 |
| 2+ Sites of infection | 2.386 | 0.708 | 8.047 | 0.16 |
| 1 Site of infection | 2.639 | 0.832 | 8.373 | 0.10 |
| No sights of infection | 1.000 | . | . | . |
| Multi-drug resistant infection | 1.806 | 0.591 | 5.513 | 0.30 |
| 3 or more pressors pre-LT | 0.755 | 0.086 | 6.638 | 0.80 |
| 2 pressors pre-LT | 1.177 | 0.536 | 2.585 | 0.69 |
| No pressors pre-LT | 1.000 | . | . | . |
| Intubated for pulmonary pathology | 2.931 | 1.246 | 6.892 | 0.014 |
| Intubated for airway protection | 1.000 | . | . | . |
| Infection duration > 14 days | 1.193 | 0.276 | 5.169 | 0.81 |
| Infection duration 7-14 days | 3.303 | 0.938 | 11.636 | 0.06 |
| Infection duration < 7 days | 3.418 | 1.059 | 11.036 | 0.04 |
| No infection | 1.000 | . | . | . |

| **Table S4:** Univariable Odds Ratios (per SD) for Continuous Measures | | | | |
|---|---|---|---|---|
| Variable | OR | Lower CL | Upper CL | p-value |
| Time on waitlist, d | 1.257 | 0.890 | 1.776 | 0.19 |
| Age, y | 1.023 | 0.704 | 1.486 | 0.90 |
| BMI | 0.977 | 0.673 | 1.420 | 0.90 |
| Days admitted prior to LT, d | 0.892 | 0.603 | 1.320 | 0.57 |
| Days admitted in ICU prior to LT, d | 1.067 | 0.746 | 1.527 | 0.72 |
| $PaO_2$:$FiO_2$ ratio at ICU admission | 0.921 | 0.631 | 1.345 | 0.67 |
| PEEP at ICU admission | 0.596 | 0.278 | 1.276 | 0.18 |
| Log WBC at ICU admission | 0.935 | 0.645 | 1.354 | 0.72 |
| Log Plts at ICU admission | 1.020 | 0.704 | 1.478 | 0.92 |
| MELD at ICU admission | 0.861 | 0.593 | 1.250 | 0.43 |
| MELD Na at ICU admission | 0.827 | 0.567 | 1.207 | 0.33 |
| CLIF-C-ACLF at ICU admission | 1.094 | 0.753 | 1.589 | 0.64 |
| $PaO_2$:$FiO_2$ ratio pre-LT | 0.830 | 0.571 | 1.205 | 0.33 |
| PEEP pre-LT | 0.989 | 0.663 | 1.473 | 0.96 |
| Base excess pre-LT | 0.601 | 0.414 | 0.872 | 0.007 |
| WBC pre-LT | 0.984 | 0.679 | 1.426 | 0.93 |
| Plt pre-LT | 0.988 | 0.683 | 1.432 | 0.95 |
| MELD pre-LT | 1.064 | 0.731 | 1.549 | 0.75 |
| MELD Na pre-LT | 1.062 | 0.729 | 1.546 | 0.75 |
| CLIF-C-ACLF pre-LT | 1.191 | 0.813 | 1.745 | 0.37 |
| DRI, per unit | 1.867 | 1.069 | 3.261 | 0.028 |
| Cold ischemia time | 0.920 | 0.626 | 1.351 | 0.67 |
| Change in MELD Na* | 1.115 | 0.753 | 1.653 | 0.59 |
| Change in MELD* | 1.083 | 0.744 | 1.576 | 0.68 |
| Change in CLIF-C-ACLF* | 0.868 | 0.572 | 1.317 | 0.51 |
| Change in WBC* | 0.829 | 0.578 | 1.188 | 0.31 |
| Change in Na* | 0.806 | 0.437 | 1.487 | 0.49 |
| Change in Plt* | 0.848 | 0.594 | 1.212 | 0.37 |
| Change in P:F ratio* | 1.071 | 0.718 | 1.597 | 0.74 |
| Percent change in MELD Na | 1.147 | 0.794 | 1.656 | 0.47 |
| Increase in MELD Na by at least 6% | 2.124 | 0.944 | 4.781 | 0.07 |
| Percent change in MELD | 1.099 | 0.769 | 1.570 | 0.61 |
| Percent change in CLIF-C-ACLF | 0.858 | 0.562 | 1.311 | 0.48 |
| Percent change WBC | 0.937 | 0.635 | 1.383 | 0.74 |
| Percent change Na | 0.802 | 0.432 | 1.487 | 0.48 |
| Percent change Plt | 0.967 | 0.646 | 1.451 | 0.87 |
| Percent change $PaO_2$:$FiO_2$ ratio | 1.096 | 0.769 | 1.561 | 0.61 |
| Decrease in $PaO_2$:$FiO_2$ ratio by at least 12% | 0.464 | 0.188 | 1.142 | 0.10 |
| *Variables denoted by "change" were calculated per SD per day from ICU admission to LT. | | | | |

| **Table S5:** Univariable odds ratios by quartiles of continuous measures | | | | |
|---|---|---|---|---|
| Variable | OR | lowercl | uppercl | p-value |
| Time on waitlist, q4 | 1.5641 | 0.50221 | 4.8713 | 0.44 |
| Time on waitlist, q3 | 1.5641 | 0.50221 | 4.8713 | 0.44 |
| Time on waitlist, q2 | 1.15384 | 0.35457 | 3.7549 | 0.81 |
| Time on waitlist, q1 | 1 | . | . | . |
| Age, q4 | 0.84416 | 0.26583 | 2.6806 | 0.77 |
| Age, q3 | 1.47887 | 0.51488 | 4.2477 | 0.47 |
| Age, q2 | 1.92309 | 0.68558 | 5.3944 | 0.21 |
| Age, q1 | 1 | . | . | . |
| BMI, q4 | 1 | 0.34384 | 2.9083 | 1.00 |
| BMI, q3 | 1.78373 | 0.6623 | 4.804 | 0.25 |
| BMI, q2 | 0.72499 | 0.23425 | 2.2438 | 0.58 |
| BMI, q1 | 1 | . | . | . |
| Days admitted prior to LT, q4 | 0.41316 | 0.15457 | 1.1044 | 0.08 |
| Days admitted prior to LT, q3 | 0.44946 | 0.17272 | 1.1696 | 0.10 |
| Days admitted prior to LT, q2 | 0.14607 | 0.04105 | 0.5198 | 0.003 |
| Days admitted prior to LT, q1 | 1 | . | . | . |
| Days admitted in ICU prior to LT, q4 | 1.52922 | 0.51263 | 4.5618 | 0.45 |
| Days admitted in ICU prior to LT, q3 | 0.60673 | 0.16558 | 2.2233 | 0.45 |
| Days admitted in ICU prior to LT, q2 | 2.77552 | 0.97317 | 7.9159 | 0.06 |
| Days admitted in ICU prior to LT, q1 | 1 | . | . | . |
| $PaO_2$:$FiO_2$ ratio at ICU admission, q4 | 0.69422 | 0.20778 | 2.3195 | 0.55 |
| $PaO_2$:$FiO_2$ ratio at ICU admission, q3 | 1.67489 | 0.58737 | 4.7759 | 0.33 |
| $PaO_2$:$FiO_2$ ratio at ICU admission, q2 | 1.48359 | 0.5132 | 4.2889 | 0.47 |
| $PaO_2$:$FiO_2$ ratio at ICU admission, q1 | 1 | . | . | . |
| PEEP at ICU admission, q4 | 0.3012 | 0.05174 | 1.7536 | 0.18 |
| PEEP at ICU admission, q1 | 1 | . | . | . |
| WBC at ICU admission, q4 | 0.65714 | 0.22842 | 1.8906 | 0.44 |
| WBC at ICU, admission, q3 | 1.12794 | 0.42625 | 2.9848 | 0.81 |
| WBC at ICU, admission, q2 | 0.65714 | 0.22842 | 1.8906 | 0.44 |
| WBC at ICU, admission, q1 | 1 | . | . | . |
| Platelets at ICU admission, q4 | 0.87927 | 0.32149 | 2.4048 | 0.80 |
| Platelets at ICU admission, q3 | 0.85518 | 0.31319 | 2.3351 | 0.76 |
| Platelets at ICU admission, q2 | 0.65714 | 0.22842 | 1.8906 | 0.44 |
| Platelets at ICU admission, q1 | 1 | . | . | . |
| CLIF-C-ACLF at LT, q4 | 1.04638 | 0.39686 | 2.7589 | 0.93 |
| CLIF-C-ACLF at LT, q3 | 0.62239 | 0.20389 | 1.8999 | 0.41 |
| CLIF-C-ACLF at LT, q2 | 1.92762 | 0.71794 | 5.1755 | 0.19 |
| CLIF-C-ACLF at LT, q1 | 1 | . | . | . |

| | | | | |
|---|---|---|---|---|
| MELD at ICU admission, q4 | 0.62441 | 0.23681 | 1.6464 | 0.34 |
| MELD at ICU admission, q3 | 0.31686 | 0.10468 | 0.9591 | 0.042 |
| MELD at ICU admission, q2 | 0.54337 | 0.20134 | 1.4664 | 0.23 |
| MELD at ICU admission, q1 | 1 | . | . | . |
| CLIF-C at ICU admission, q4 | 1.12794 | 0.42625 | 2.9848 | 0.81 |
| CLIF-C at ICU admission, q3 | 0.55473 | 0.18596 | 1.6548 | 0.29 |
| CLIF-C at ICU admission, q2 | 0.76516 | 0.27356 | 2.1402 | 0.61 |
| CLIF-C at ICU admission, q1 | 1 | . | . | . |
| $PaO_2$:$FiO_2$ ratio at LT, q4 | 0.72632 | 0.24781 | 2.1288 | 0.56 |
| $PaO_2$:$FiO_2$ ratio at LT, q3 | 0.62948 | 0.20697 | 1.9145 | 0.41 |
| $PaO_2$:$FiO_2$ ratio at LT, q2 | 1.50852 | 0.57229 | 3.9764 | 0.41 |
| $PaO_2$:$FiO_2$ ratio at LT, q1 | 1 | . | . | . |
| PEEP at LT, q4 | 0.88093 | 0.2014 | 3.8532 | 0.87 |
| PEEP at LT, q1 | 1 | . | . | . |
| Base excess at LT, q4 | 0.18744 | 0.06102 | 0.5758 | 0.004 |
| Base excess at LT, q3 | 0.23636 | 0.07602 | 0.7349 | 0.012 |
| Base excess at LT, q2 | 0.38314 | 0.14961 | 0.9812 | 0.046 |
| Base excess at LT, q1 | 1 | . | . | . |
| WBC at LT, q4 | 1.30691 | 0.46725 | 3.6555 | 0.61 |
| WBC at LT, q3 | 1.43139 | 0.52003 | 3.9399 | 0.49 |
| WBC at LT, q2 | 0.72499 | 0.23425 | 2.2438 | 0.58 |
| WBC at LT, q1 | 1 | . | . | . |
| Platelets at LT, q4 | 1.14996 | 0.40271 | 3.2837 | 0.79 |
| Platelets at LT, q3 | 1.00447 | 0.35441 | 2.8469 | 0.99 |
| Platelets at LT, q2 | 1.14996 | 0.40271 | 3.2837 | 0.79 |
| Platelets at LT, q1 | 1 | . | . | . |
| MELD at LT, q4 | 1 | 0.34384 | 2.9083 | 1.00 |
| MELD at LT, q3 | 0.97333 | 0.33516 | 2.8266 | 0.96 |
| MELD at LT, q2 | 1.47412 | 0.5346 | 4.0647 | 0.45 |
| MELD at LT, q1 | 1 | . | . | . |
| CLIF-C-ACLF, q4 | 1.80267 | 0.6043 | 5.3775 | 0.29 |
| CLIF-C-ACLF, q3 | 1.54161 | 0.50912 | 4.668 | 0.44 |
| CLIF-C-ACLF, q2 | 1.80267 | 0.6043 | 5.3775 | 0.29 |
| CLIF-C-ACLF, q1 | 1 | . | . | . |
| Cold ischemia time, q4 | 0.84433 | 0.29517 | 2.4152 | 0.75 |
| Cold ischemia time, q3 | 1.13928 | 0.41353 | 3.1387 | 0.80 |
| Cold ischemia time, q2 | 0.62798 | 0.20583 | 1.9159 | 0.41 |
| Cold ischemia time, q1 | 1 | . | . | . |
| MELD change ICU to LT, q4 | 1.92309 | 0.68558 | 5.3944 | 0.21 |
| MELD change ICU to LT, q3 | 1.13333 | 0.37978 | 3.382 | 0.82 |
| MELD change ICU to LT, q2 | 1.16438 | 0.38963 | 3.4797 | 0.79 |

| | | | | |
|---|---|---|---|---|
| MELD change ICU to LT, q1 | 1 | . | . | . |
| CLIF-C ACLF change ICU to LT, q4 | 1.28281 | 0.47593 | 3.4577 | 0.62 |
| CLIF-C ACLF change ICU to LT, q3 | 0.5203 | 0.16364 | 1.6544 | 0.27 |
| CLIF-C ACLF change ICU to LT, q2 | 1.13731 | 0.41583 | 3.1105 | 0.80 |
| CLIF-C ACLF change ICU to LT, q1 | 1 | . | . | . |
| WBC change ICU to LT, q4 | 1.33802 | 0.45725 | 3.9154 | 0.60 |
| WBC change ICU to LT, q3 | 1.92309 | 0.68558 | 5.3943 | 0.21 |
| WBC change ICU to LT, q2 | 1 | 0.32587 | 3.0687 | 1.00 |
| WBC change ICU to LT, q1 | 1 | . | . | . |
| Na change ICU to LT, q4 | 0.65714 | 0.22842 | 1.8906 | 0.44 |
| Na change ICU to LT, q3 | 0.52381 | 0.16614 | 1.6515 | 0.27 |
| Na change ICU to LT, q2 | 1.18286 | 0.46225 | 3.0268 | 0.73 |
| Na change ICU to LT, q1 | 1 | . | . | . |
| Platelets change ICU to LT, q4 | 1.26375 | 0.48346 | 3.3034 | 0.63 |
| Platelets change ICU to LT, q3 | 0.85518 | 0.31319 | 2.3351 | 0.76 |
| Platelets change ICU to LT, q2 | 0.36502 | 0.10911 | 1.2211 | 0.10 |
| Platelets change ICU to LT, q1 | 1 | . | . | . |
| $PaO_2$:$FiO_2$ ratio change ICU to LT, q4 | 0.76259 | 0.27075 | 2.1479 | 0.61 |
| $PaO_2$:$FiO_2$ ratio change ICU to LT, q3 | 0.76259 | 0.27075 | 2.1479 | 0.61 |
| $PaO_2$:$FiO_2$ ratio change ICU to LT, q2 | 0.65392 | 0.22579 | 1.8939 | 0.43 |
| $PaO_2$:$FiO_2$ ratio change ICU to LT, q1 | 1 | . | . | . |
| Percent change MELD ICU to LT, q4 | 1.92309 | 0.68558 | 5.3944 | 0.21 |
| Percent change MELD ICU to LT, q3 | 0.97403 | 0.31783 | 2.985 | 0.96 |
| Percent change MELD ICU to LT, q2 | 1.33802 | 0.45725 | 3.9154 | 0.60 |
| Percent change MELD ICU to LT, q1 | 1 | . | . | . |
| Percent change CLIF-C-ACLF ICU to LT, q4 | 1.28281 | 0.47593 | 3.4577 | 0.62 |
| Percent change CLIF-C-ACLF ICU to LT, q3 | 0.6309 | 0.20805 | 1.9132 | 0.42 |
| Percent change CLIF-C-ACLF ICU to LT, q2 | 1 | 0.35917 | 2.7842 | 1.00 |
| Percent change CLIF-C-ACLF ICU to LT, q1 | 1 | . | . | . |
| Percent change WBC ICU to LT, q4 | 1.52174 | 0.52894 | 4.378 | 0.44 |
| Percent change WBC ICU to LT, q3 | 1.92309 | 0.68558 | 5.3943 | 0.21 |
| Percent change WBC ICU to LT, q2 | 0.84416 | 0.26583 | 2.6806 | 0.77 |
| Percent change WBC ICU to LT, q1 | 1 | . | . | . |
| Percent change bilirubin ICU to LT, q4 | 2.35294 | 0.73260 | 7.55705 | 0.15 |
| Percent change bilirubin ICU to LT, q3 | 2.05714 | 0.62984 | 6.71886 | 0.23 |
| Percent change bilirubin ICU to LT, q2 | 2.66666 | 0.84163 | 8.44916 | 0.10 |
| Percent change bilirubin ICU to LT, q1 | 1 | . | . | . |
| Percent change INR ICU to LT, q4 | 0.90909 | 0.34138 | 2.42092 | 0.85 |
| Percent change INR ICU to LT, q3 | 0.79481 | 0.29257 | 2.15919 | 0.65 |
| Percent change INR ICU to LT, q2 | 0.39627 | 0.12512 | 1.25503 | 0.12 |
| Percent change INR ICU to LT, q1 | 1 | . | . | . |

| | | | | |
|---|---|---|---|---|
| Percent change Na, ICU to LT, q4 | 0.65714 | 0.22842 | 1.8906 | 0.44 |
| Percent change Na, ICU to LT, q3 | 0.52381 | 0.16614 | 1.6515 | 0.27 |
| Percent change Na, ICU to LT, q2 | 1.18286 | 0.46225 | 3.0268 | 0.73 |
| Percent change Na, ICU to LT, q1 | 1 | . | . | . |
| Percent change platelets, ICU to LT, q4 | 0.87927 | 0.32149 | 2.4048 | 0.80 |
| Percent change platelets, ICU to LT, q3 | 0.85518 | 0.31319 | 2.3351 | 0.76 |
| Percent change platelets, ICU to LT, q2 | 0.65714 | 0.22842 | 1.8906 | 0.44 |
| Percent change platelets, ICU to LT, q1 | 1 | . | . | . |
| Percent change $PaO_2$:$FiO_2$ ratio ICU to LT, q4 | 0.77676 | 0.286 | 2.1096 | 0.62 |
| Percent change $PaO_2$:$FiO_2$ ratio ICU to LT, q3 | 0.48779 | 0.16464 | 1.4452 | 0.20 |
| Percent change $PaO_2$:$FiO_2$ ratio ICU to LT, q2 | 0.57868 | 0.20255 | 1.6533 | 0.31 |
| Percent change $PaO_2$:$FiO_2$ ratio ICU to LT, q1 | 1 | . | . | . |

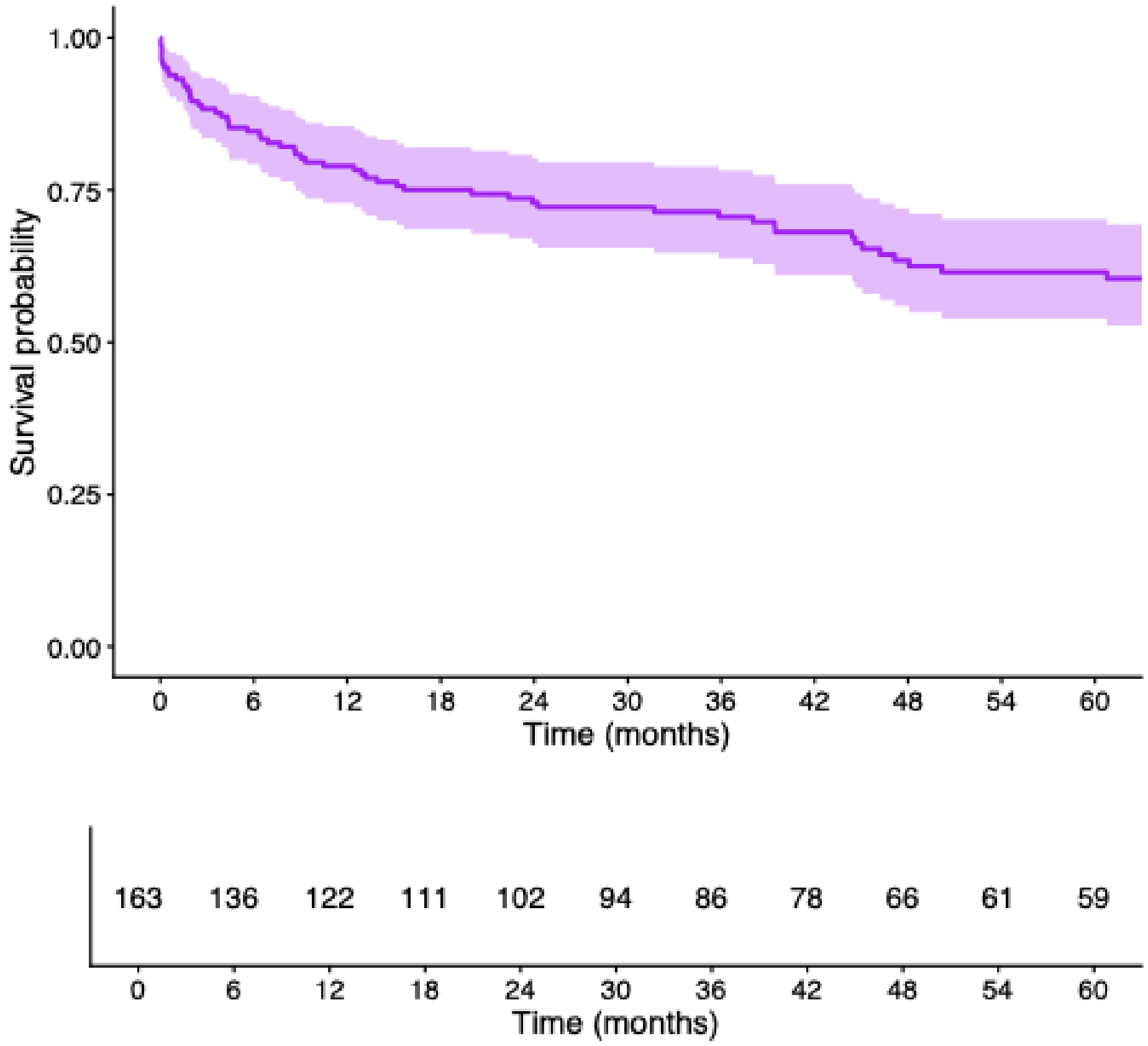


**Fig. S1**: Overall post-transplant survival in extreme ACLF-3 patients excluding those receiving a simultaneous liver-kidney transplant (n=14).

49 Spadina Ave. Suite 200
Toronto ON M5V 2J1 Canada
www.biorender.com

# Confirmation of Publication and Licensing Rights - Open Access

**December 11th, 2025**

| | |
|---|---|
| ***Subscription Type:*** | *Postdoc Plan - Academic* |
| ***Agreement number:*** | *QW293RUI0C* |
| ***Publisher Name:*** | *JHEP Reports* |
| ***Figure Title:*** | *Graphical Abstract* |
| ***Citation to Use:*** | *Created in BioRender. Melehy, A. (2026) https://BioRender.com/t59ytz1* |

To whom this may concern,

This document ("Confirmation") hereby confirms that Science Suite Inc. dba BioRender ("BioRender") has granted the following BioRender user: Andrew Melehy ("User") a BioRender Academic Publication License in accordance with BioRender's Terms of Service and Academic License Terms ("License Terms") to permit such User to do the following on the condition that all requirements in this Confirmation are met:

1) publish their Completed Graphics created in the BioRender Services containing both User Content and BioRender Content (as both are defined in the License Terms) in publications (journals, textbooks, websites, etc.); and
2) sublicense such Completed Graphics under "open access" publication sublicensing models such as CC-BY 4.0 and more restrictive models, so long as the conditions set forth herein are fully met.

Requirements of User:

1) All Completed Graphics to be published in any publication (journals, textbooks, websites, etc.) must be accompanied by the following citation either as a caption, footnote or reference for each figure that includes a Completed Graphic:
"Created in BioRender. Melehy, A. (2026) https://BioRender.com/t59ytz1 ".
2) All terms of the License Terms including all Prohibited Uses are fully complied with. E.g. For Academic License Users, no commercial uses (beyond publication in journals, textbooks or websites) are permitted without obtaining or switching to a BioRender Industry Plan.
3) A Reader (defined below) may request that the User allow their figure to be a public template for Readers to view, copy, and modify the figure. It is up to the User to determine what level of access to grant.

Open-Access Journal Readers:

Open-Access journal readers ("Reader") who wish to view and/or re-use a particular Completed Graphic in an Open-Access journal subject to CC-BY sublicensing may do so by clicking on the URL link in the applicable citation for the subject Completed Graphic.

The re-use/modification options below are available after the Reader requests the User to adapt their
